# Real-World Evidence of a Hospital-Linked Digital Health App for the Control of Hypertension and Diabetes Mellitus in South Korea: Nationwide Multicenter Study

**DOI:** 10.2196/48332

**Published:** 2023-08-21

**Authors:** Sangil Park, Ho Geol Woo, Soeun Kim, Sunyoung Kim, Hyunjung Lim, Dong Keon Yon, Sang Youl Rhee

**Affiliations:** 1 Department of Neurology Kyung Hee University Medical Center Kyung Hee University College of Medicine Seoul Republic of Korea; 2 Center for Digital Health, Medical Science Research Institute Kyung Hee University Medical Center Kyung Hee University College of Medicine Seoul Republic of Korea; 3 Department of Family Medicine Kyung Hee University Medical Center Kyung Hee University College of Medicine Seoul Republic of Korea; 4 Department of Medical Nutrition Kyung Hee University Seoul Republic of Korea

**Keywords:** hypertension, blood pressure, diabetes, glucose, digital health technology, effectiveness, application, blood glucose, systolic, diastolic, management, consumer, cost, monitoring

## Abstract

**Background:**

Digital health care apps have been widely used for managing chronic conditions such as diabetes mellitus and hypertension, providing promising prospects for enhanced health care delivery, increased patient engagement, and improved self-management. However, the impact of integrating these apps within hospital systems for managing such conditions still lacks conclusive evidence.

**Objective:**

We aimed to investigate the real-world effectiveness of using hospital-linked digital health care apps in lowering blood pressure (BP) and blood glucose levels in patients with hypertension and diabetes mellitus.

**Methods:**

Nationwide multicenter data on demographic characteristics and the use of a digital health care app from 233 hospitals were collected for participants aged 20 to 80 years in South Korea between August 2021 and June 2022. We divided the participants into 2 groups: 1 group consisted of individuals who exclusively used the digital health app (control) and the other group used the hospital-linked digital health app. All the patients participated in a 12-week digital health care intervention. We conducted a comparative analysis to assess the real-world effectiveness of the hospital-linked digital health app. The primary outcome was the differences in the systolic blood pressure (SBP), diastolic blood pressure (DBP), fasting blood glucose (FBG) level, and postprandial glucose (PPG) level between baseline and 12 weeks.

**Results:**

A total of 1029 participants were analyzed for the FBG level, 527 participants were analyzed for the PPG level, and 2029 participants for the SBP and DBP were enrolled. After 12 weeks, a hospital-linked digital health app was found to reduce SBP (−5.4 mm Hg, 95% CI −7.0 to −3.9) and DBP (−2.4 mm Hg, 95% CI −3.4 to −1.4) in participants without hypertension and FBG level in all participants (those without diabetes, −4.4 mg/dL, 95% CI −7.9 to −1.0 and those with diabetes, −3.2 mg/dL, 95% CI −5.4 to −1.0); however, there was no statistically significant difference compared to the control group (using only digital health app). Specifically, participants with diabetes using a hospital-linked digital health app demonstrated a significant decrease in PPG after 12 weeks (−10.9 mg/dL, 95% CI −31.1 to −5.3) compared to those using only a digital health app (*P*=.006).

**Conclusions:**

Hospital-linked digital interventions have greatly improved glucose control for diabetes compared with using digital health technology only. These hospital-linked digital health apps have the potential to offer consumers and health care professionals cost-effective support in decreasing glucose levels when used in conjunction with self-monitoring.

## Introduction

Diabetes mellitus is a comorbid disease frequently complicating hypertension, which greatly increases its overall morbidity and mortality [1]. In addition, hypertension is a significant contributor to the risk of cardiovascular disease and is more common in those with diabetes mellitus [2]. Cardiovascular and renal vascular disorders are 2 of the most expensive consequences of diabetes in terms of personal suffering and societal health care expenses [1-3]. Therefore, an essential part of the comprehensive clinical management of patients with diabetes is the diagnosis and treatment of elevated blood pressure (BP) and blood glucose levels.

Continuously lowering BP, controlling blood lipid levels, and controlling blood glucose levels can all reduce the risk of problems related to diabetes [1,2]. The American Diabetes Association currently recommends that the majority of adults with diabetes achieve a glycated hemoglobin level of <7.0%, BP measurement of <140/90 mm Hg (<130/90 for patients with an increased cardiovascular risk), and low-density lipoprotein cholesterol level of <100 mg/dL [4]. Developing knowledge, skills, and abilities necessary for the successful self-management of interventions for diabetes through diabetes-specific personality education and support, which is defined as an interactive and ongoing process, has been proven to be successful [4]. Similarly, self-monitoring combined with education or counseling may help patients with hypertension improve their BP management and drug compliance [5].

Globally, the use of information and communication technologies in health care is expanding quickly [6]. By bridging the gap between qualified medical treatment and patient self-management, digital health technology has been approved as a way to increase access, effectiveness, and quality of life [7]. Nevertheless, the outcomes have been inconsistent. According to 2 reviews, telemedicine effectively lowers BP, whereas other reviews have not consistently demonstrated this result [8,9]. Digital health care apps have been widely used for managing chronic conditions such as diabetes mellitus and hypertension, providing promising prospects for enhanced health care delivery, increased patient engagement, and improved self-management. However, the impact of integrating these apps within hospital systems for managing such conditions still lacks conclusive evidence [10]. Thus, we aimed to investigate the real-world effectiveness of using hospital-linked digital health care apps to lower BP and blood glucose levels in patients with hypertension and diabetes mellitus.

## Methods

### Study Population and Data Sources

The participants who were aged 20 to 80 years and used a digital health app (WellCheck) responded to questions regarding their histories of high BP and diabetes and subsequently entered their BP and blood glucose levels (fasting and postprandial) within 1 week. The patients were encouraged to install a digital health app and establish a connection with the hospital to enable physicians to monitor their health. However, those patients who voluntarily opted to exclusively use the digital health app were considered as the control group. Additionally, the group using the digital health app connected to the hospital was designated as the observation group for reporting the observed outcomes. The study was conducted between August 2021 and June 2022. BP and blood glucose levels were measured in the first week to create a baseline value, and follow-up was performed until the 12th week to evaluate the improvement in those values. Three separate sets of BP and blood glucose analyses were performed during the 12 weeks. Cases where the measured BP and blood glucose levels differed from the common reference values were eliminated from the study. Finally, 1029 participants (those using only digital health app: n=169 vs those using hospital-linked digital health app: n=860) were analyzed for the blood glucose level before meals, 527 participants (those using only digital health app: n=76 vs those using hospital-linked digital health app: n=451) were analyzed for the blood glucose level after meals, and 2029 participants (those using only digital health app: n=215 vs those using hospital-linked digital health app: n=1814) were analyzed for BP were enrolled in the study.

### Ethics Approval

The study protocol was approved by the institutional review board of Kyung Hee University (KHSIRB-22-570), and the need for written informed consent was waived because of the use of a routinely collected health data set.

### WellCheck App Device

Hospital-linked digital health app (WellCheck) is different from other simple digital health apps for connection between “application for patients” and “web and electronic medical record for doctors” to manage diabetes and hypertension. Using the hospital-linked digital health app, doctors conveniently check the health status of patients and manage it for “web and electronic medical record.” Hospital-linked digital health app enables users to record BP and blood glucose and their specific health concerns and alarms user if BP and blood glucose are not recorded. The data can then be sent to doctors for analysis and monitoring. If the user records very high or low levels of BP or blood sugar compared to the target of them, doctors can send feedback messages to manage it based on their judgment. Through this, doctors can manage patients and monitor at-risk patients separately. Also, users can view how effectively their levels are controlled in relation to the targets in the chosen ways among graphs, score tables, and lists. Additionally, the National Health Insurance Service of South Korea allows patients to obtain the findings of their comprehensive health assessment for their entire lifetime, present them to the attending physician, and receive a feedback message from their doctor [11,12]. Through this, the management of BP or blood sugar was greatly improved. Therefore, we performed the analysis among patients using only a digital health app versus those using a hospital-linked digital health app (Multimedia Appendix 1).

### Definitions and Outcomes

According to the 2020 American College of Cardiology and American Heart Association hypertension guidelines, the BP categories are hypertension (systolic BP [SBP] ≥130 mm Hg or diastolic BP [DBP] ≥80 mm Hg) or not (SBP<130 mm Hg and DBP<80 mm Hg) [13]. It was recommended that the patients performed at least 2 readings and entered the average value in order to accurately measure their BP [14]. According to the 2020 version of the Standards of Medical Care in Diabetes diagnostic criteria, diabetes diagnosis was based on glycated hemoglobin ≥6.5%, fasting blood glucose (FBG) ≥126 mg/dL, or random plasma glucose ≥200 mg/dL with classic symptoms of hyperglycemia or hyperglycemic crisis [15]. Our population was divided into diagnostic groups according to the condition (diabetes or hypertension) or not [15].

SBP and DBP were divided, but a pair of SBP and DBP data were used to measure improvement in approaching the objective range. We also measured the FBG and postprandial glucose (PPG) levels. The primary outcome was the difference in the SBP, DBP, FBG, and PPG levels between baseline and 12 weeks. The secondary outcomes included variations in the baseline SBP, DBP, FBG, and PPG levels at 4 and 8 weeks.

### Statistical Analyses

We performed 1:3 propensity score matching three times to reduce potential confounding effects [16,17]: (1) cohort A1 and A2: age, sex, hypertension status (A1: normal or prehypertension, A2: hypertension), and baseline BP; (2) cohort B1 and B2: age, sex, diabetes status (B1: normal or prehypertension, B2: hypertension), and baseline FBG; and (3) cohort C1 and C2: age, sex, diabetes status (C1: normal or prediabetes, C2: diabetes), and baseline PPG. We then assessed the mean difference of BP and blood glucose levels at 4, 8, and 12 weeks compared to the first week to evaluate their improvement. Analyses of covariance were used to compare different groups while adjusting for baseline values and demographic factors (identified by stepwise removal at *P*<.10 [2-tailed]). First, participant comparisons of those who completed data entry were conducted. By examining 2-way interactions in the analyses of covariance models between the hospital linkage and nonlinkage groups, SAS (version 9.4; SAS Institute, Inc) and SPSS (version 26.0; IBM Corp) were used to perform all of the analyses [18,19]. Statistical significance was defined as a 2-sided *P* value of <.05.

## Results

### Overview

A total of 2209 participants recorded their BP, 1029 participants recorded their FBG levels, and 527 participants recorded their PPG levels from August 2021 to July 2022. Multimedia Appendix 1 categorizes and presents the baseline characteristics of the participants in terms of BP and blood glucose levels before and after propensity score matching based on whether they were registered with their hospital. There was more prominence in male participants who recorded BP (nonlinked: 137/215, 63%; linked: 1057/1814, 58%), FBG (nonlinked: 106/169, 62%; linked: 492/860, 57%), and PPG (nonlinked: 46/76, 60%; linked: 226/451, 50%). Hypertension (nonlinked: 158/215, 73%; linked: 1540/1814, 84%) and diabetes (FBG, nonlinked: 131/169, 77%; FBG, linked: 750/860, 87%; PPG, nonlinked: 61/76, 80%; PPG, linked: 379/451, 84%) were highly prevalent among the participants.

### Blood Pressure

**Figure 1:**
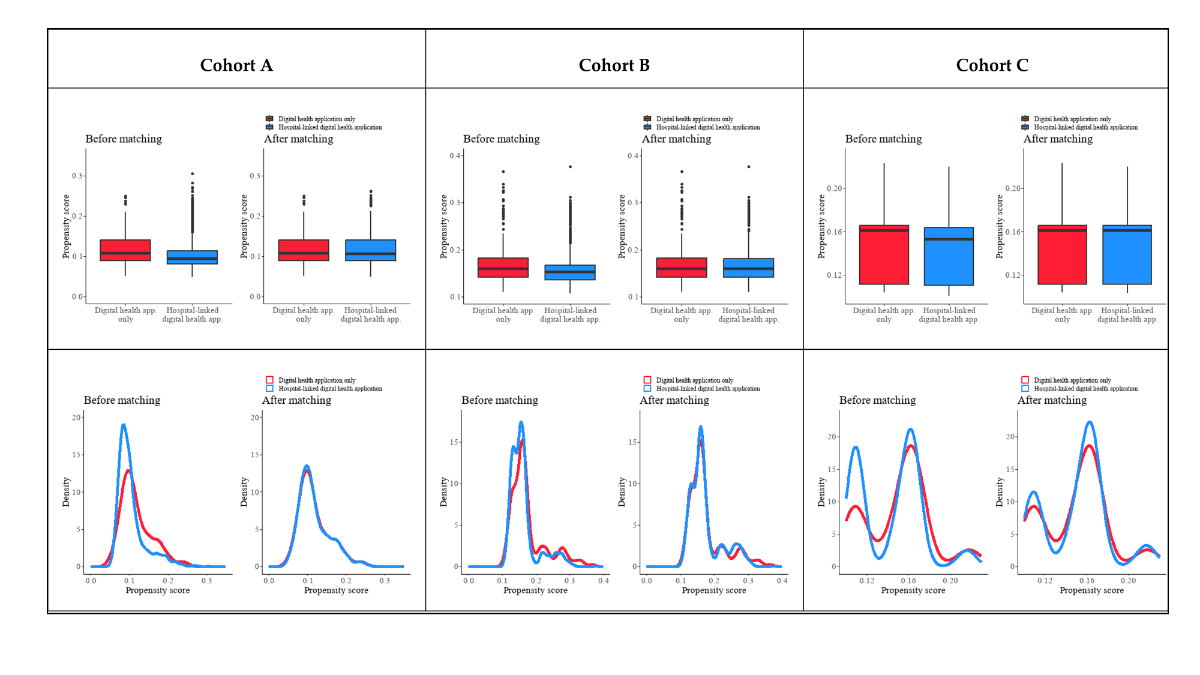
Density and box plot of propensity scores before and after matching in cohort A, B, and C. For a higher-resolution version of this figure, see Multimedia Appendix 2.

In propensity score-matched cohort A (Figure 1), all participants without hypertension group using the hospital-linked digital health app exhibited a significant decrease in SBP, with actual mean differences of −4.6 mm Hg and adjusted mean differences of −4.9 mm Hg (95% CI −6.4 to −3.5) after 4 weeks, −5.0 mm Hg and −5.3 mm Hg (95% CI −6.8 to −3.8) after 8 weeks, and −5.4 mm Hg and −5.4 mm Hg (95% CI −7.0 to −3.9) after 12 weeks compared with the baseline, respectively. However, there was no difference in the reduced SBP during weeks 4-12 in the participants with and without hypertension group using the hospital-linked digital health app (those without hypertension: −1.9, 95% CI −5.0 to 1.2 after 12 weeks; those with hypertension: −0.8, 95% CI −2.6 to 1.0 after 12 weeks) (Table 1).

All participants without hypertension group using the hospital-linked digital health app exhibited a significant decrease in DBP, with actual mean differences of −2.3 mm Hg and adjusted mean differences of −1.5 mm Hg (95% CI −2.5 to −0.5) after 4 weeks, −2.8 mm Hg and −2.6 mm Hg (95% CI −3.7 to −1.5) after 8 weeks, and −2.6 mm Hg and −2.4 mm Hg (95% CI −3.4 to −1.4) after 12 weeks, respectively. However, there was no difference in the reduced DBP during weeks 4-12 in the participants with and without hypertension group using the hospital-linked digital health app (those without hypertension: 0.2, 95% CI −1.8 to 2.2 after 12 weeks; those with hypertension: −0.7, 95% CI 2.0 to 0.6 after 12 weeks; and all *P*≥.05) (Table 2).

**Table 1:** Comparison of the systolic blood pressure results by hypertension status between the participants in the cohort using only the digital health app and those using the hospital-linked digital health app.

Participants					0 weeks			4 weeks			8 weeks			12 weeks
**Patients without hypertension (normal + prehypertension)**														
	**Digital health app only**													
		SBP^a^ (mm Hg), mean (95% CI)	123.8 (121.2 to 126.5)			121.2 (117.7 to 124.6)			121.2 (117.7 to 124.8)			120.3 (117.4 to 123.2)		
		Crude mean difference (95% CI)	Reference			−2.2 (−4.7 to 0.3)			−*3.3* (−*5.9 to -0.8*)^b^			−*3.5* (−*5.8 to* −*1.2*)		
		Adjusted mean difference^c^ (95% CI)	Reference			−2.4 (−5.0 to 0.2)			−*3.5* (−*6.2 to* −*0.7*)			−*3.5* (−*6.2 to* −*0.8*)		
	**Hospital-linked digital health app**													
		SBP (mm Hg), mean (95% CI)	124.1 (122.4 to 125.8)			119.5 (118.0 to 121.1)			119.1 (117.5 to 120.6)			118.7 (117.1 to 120.2)		
		Crude mean difference (95% CI)	Reference			−*5.0* (−*6.5 to* −*3.5*)			−*5.3* (−*6.8 to* −*3.8*)			−*5.4* (−*7.0 to* −*3.8*)		
		Adjusted mean difference (95% CI)	Reference			−*4.9* (−*6.4 to* −*3.5*)			−*5.3* (−*6.8 to* −*3.8*)			−*5.4* (−*7.0 to* −*3.9*)		
	Comparison of the adjusted mean differences between the 2 groups (95% CI); *P* value			N/A^d^			−2.5 (−5.3 to 0.3); *P*=.09			−1.8 (−4.9 to 1.3); *P*=.25			−1.9 (−5.0 to 1.2); *P*=.23	
**Patients with hypertension**														
	**Digital health app only**													
		SBP (mm Hg), mean (95% CI)	121.3 (119.6 to 123.0)			121.3 (119.4 to 123.2)			121.5 (119.4 to 123.5)			121.8 (120.3 to 123.3)		
		Crude mean difference (95% CI)	Reference			0.5 (−1.2 to 2.2)			0.2 (−2.0 to 2.3)			0.5 (−1.2 to 2.2)		
		Adjusted mean difference (95% CI)	Reference			0.5 (−0.9 to 1.9)			0.1 (−1.6 to 1.9)			0.5 (−1.1 to 2.0)		
	**Hospital-linked digital health app**													
		SBP (mm Hg), mean (95% CI)	121.5 (120.6 to 122.4)			120.8 (120.0 to 121.7)			120.5 (119.6 to 121.3)			121.2 (120.4 to 122.1)		
		Crude mean difference (95% CI)	Reference			−0.3 (−1.1 to 0.4)			−0.3 (−1.2 to 0.5)			−0.3 (−1.1 to 0.6)		
		Adjusted mean difference (95% CI)	Reference			−0.3 (−1.2 to 0.5)			−0.3 (−1.2 to 0.7)			−0.3 (−1.1 to 0.6)		
	Comparison of the adjusted mean differences between the 2 groups (95% CI); *P* value			N/A			−0.8 (−2.4 to 0.8); *P*=.32			−0.4 (−2.4 to 1.6); *P*=.64			−0.8 (−2.6 to 1.0); *P*=.42	

^a^SBP: systolic blood pressure.

^b^Values in italic indicate a significant difference (*P*<.05).

^c^The adjusted model was adjusted for age, sex, and baseline SBP.

^d^N/A: not applicable.

**Table 2:** Comparison of the diastolic blood pressure results by hypertension status between the participants in the cohort using only the digital health app and those using the hospital-linked digital health app.

Participants					0 weeks			4 weeks			8 weeks			12 weeks
**Patients without hypertension (normal + prehypertension)**														
	**Digital health app only**													
		DBP^a^ (mm Hg), mean (95% CI)	79.1 (77.9 to 80.3)			78.6 (77.4 to 79.9)			78.3 (76.9 to 79.6)			78.8 (77.7 to 79.9)		
		Crude mean difference (95% CI)	Reference			−*1.7* (−*3.3 to* −*0.1*)^b^			−*2.5* (−*4.5 to* −*0.6*)			−*2.7* (−*4.3 to* −*1.0*)		
		Adjusted mean difference^c^ (95% CI)	Reference			−1.7 (−3.5 to 0.1)			−*2.6* (−*4.6 to* −*0.6*)			−*2.6* (−*4.4 to* −*0.9*)		
	**Hospital-linked digital health app**													
		DBP (mm Hg), mean (95% CI)	79.9 (80.1 to 81.6)			77.6 (77.9 to 79.3)			77.1 (77.4 to 78.8)			77.3 (77.6 to 78.9)		
		Crude mean difference (95% CI)	Reference			−*1.5* (−*2.5 to* −*0.5*)			−*2.6* (−*3.8 to* −*1.5*)			−*2.4* (−*3.4 to* −*1.3*)		
		Adjusted mean difference (95% CI)	Reference			−*1.5* (−*2.5 to* −*0.5*)			−*2.6* (−*3.7 to* −*1.5*)			−*2.4* (−*3.4 to* −*1.4*)		
	Comparison of the adjusted mean differences between the 2 groups (95% CI); *P* value			N/A^d^			0.2 (−1.9 to 2.3); *P*=.84			0.0 (−2.3 to 2.3); *P*=.97			0.2 (−1.8 to 2.2); *P*=.81	
**Patients with hypertension**														
	**Digital health app only**													
		DBP (mm Hg), mean (95% CI)	78.7 (77.4 to 80.1)			78.7 (77.3 to 80.1)			78.5 (77.0 to 80.0)			78.9 (77.6 to 80.1)		
		Crude mean difference (95% CI)	Reference			0.3 (−0.8 to 1.4)			−0.2 (−1.6 to 1.2)			0.1 (−1.0 to 1.3)		
		Adjusted mean difference (95% CI)	Reference			0.3 (−0.8 to 1.4)			−0.2 (−1.5 to 1.1)			0.2 (−1.0 to 1.3)		
	**Hospital-linked digital health app**													
		DBP (mm Hg), mean (95% CI)	78.8 (78.0 to 79.6)			78.5 (77.7 to 79.2)			78.2 (77.4 to 79.0)			78.4 (77.7 to 79.1)		
		Crude mean difference (95% CI)	Reference			−0.2 (−0.9 to 0.4)			−0.5 (−1.2 to 0.2)			−0.5 (−1.1 to 0.2)		
		Adjusted mean difference (95% CI)	Reference			−0.3 (−0.9 to 0.4)			−0.5 (−1.2 to 0.2)			−0.5 (−1.1 to 0.2)		
	Comparison of the adjusted mean differences between the 2 groups (95% CI); *P* value			N/A			−0.6 (−1.9 to 0.7); *P*=.39			−0.3 (−1.8 to 1.2); *P*=.66			−0.7 (2.0 to 0.6); *P*=.38	

^a^DBP: diastolic blood pressure.

^b^Values in italic indicate a significant difference (*P*<.05).

^c^The adjusted model was adjusted for age, sex, and baseline DBP.

^d^N/A: not applicable.

### Blood Glucose

In propensity score-matched cohort B, the participants without diabetes using only the digital health app exhibited a significant decrease in FBG, with actual mean differences of −4.5 mg/dL and adjusted mean differences of −4.4 mg/dL (95% CI −7.9 to −1.0) after 12 weeks compared with the baseline. Similar trends were observed in those in the diabetes group using the hospital-linked digital health app, who exhibited adjusted mean differences of −3.2 mg/dL (95% CI −5.4 to −1.0) after 12 weeks compared with the baseline (Table 3).

**Figure 2:**
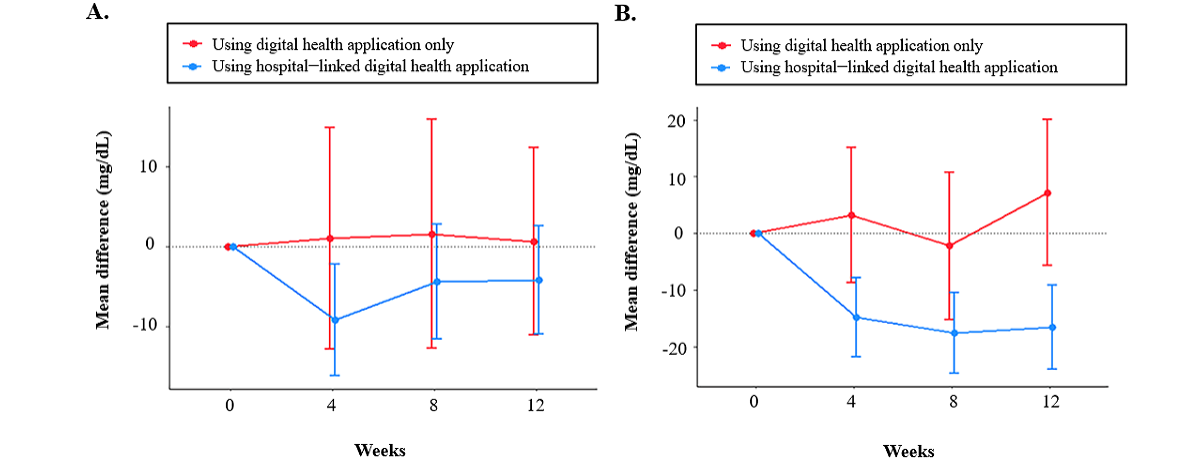
Comparison of the postprandial blood glucose levels by diabetes status among (A) those without diabetes and (B) with diabetes.

In propensity score-matched cohort C, the participants in the diabetes group using the hospital-linked digital health app exhibited a marked decrease in PPG, with actual mean differences of −11.5 mg/dL and adjusted mean differences of −10.9 mg/dL (95% CI −16.8 to −5.0) after 4 weeks, −11.5 mg/dL and −9.7 mg/dL (95% CI −16.0 to −3.3) after 8 weeks, and −11.0 mg/dL and −10.9 mg/dL (95% CI −17.4 to −4.7) after 12 weeks compared with the baseline, respectively. Specifically, participants with diabetes using a hospital-linked digital health app demonstrated a significant decrease in PPG after 12 weeks compared to those using only a digital health app (−18.2 mg/dL, 95% CI −31.1 to −5.3; *P*=.006) (Table 4 and Figure 2).

**Table 3:** Comparison of the fasting blood glucose levels by diabetes status between the participants in the cohort using only the digital health app and those using the hospital-linked digital health app.

Participants					0 weeks			4 weeks			8 weeks			12 weeks
**Patients without diabetes (normal + prediabetes)**														
	**Digital health app only**													
		FBG^a^ (mg/dL), mean (95% CI)		109.2 (105.1 to 111.4)			109.3 (104.4 to 112.2)			108.1 (102.4 to 111.7)			107.6 (102.1 to 111.1)	
		Crude mean difference (95% CI)		Reference			−0.9 (−3.5 to 1.8)			−1.8 (−6.5 to 2.8)			−1.6 (−5.4 to 2.1)	
		Adjusted mean difference^b^ (95% CI)		Reference			−0.7 (−6.0 to 4.6)			−2.0 (−8.7 to 4.7)			−1.6 (−7.5 to 4.2)	
	**Hospital-linked digital health app**													
		FBG (mg/dL), mean (95% CI)		110.9 (110.3 to 119.4)			109.7 (110.1 to 117.2)			108.5 (108.3 to 116.6)			106.4 (107.4 to 113.5)	
		Crude mean difference (95% CI)		Reference			−1.6 (−5.2 to 1.9)			−3.0 (−7.2 to 1.2)			−*4.4* (−*8.2 to* −*0.7*)^c^	
		Adjusted mean difference (95% CI)		Reference			−1.7 (−4.8 to 1.4)			−2.9 (−6.8 to 0.9)			−*4.4* (−*7.9 to* −*1.0*)	
	Comparison of the adjusted mean differences between the 2 groups (95% CI); *P* value			N/A^d^			−1.0 (−7.1 to 5.1); *P*=.76			−0.9 (−8.6 to 6.8); *P*=.80			−2.8 (−8.6 to 3.0); *P*=.41	
**Patients with diabetes**														
	**Digital health app only**													
		FBG (mg/dL), mean (95% CI)		123.4 (119.5 to 127.3)			121.5 (116.7 to 126.2)			118.0 (114.7 to 121.3)			117.2 (113.5 to 120.9)	
		Crude mean difference (95% CI)		Reference			−0.8 (−4.4 to 2.8)			−*6.2* (−*10.0 to* −*2.3*)			−*6.2* (−*10.1 to* −*2.4*)	
		Adjusted mean difference (95% CI)		Reference			−0.8 (−4.4 to 2.7)			−*6.1* (−*10.0 to* −*2.2*)			−*6.2* (−*10.0 to* −*2.5*)	
	**Hospital-linked digital health app**													
		FBG (mg/dL), mean (95% CI)		122.4 (120.2 to 124.6)			121.1 (118.0 to 122.2)			120.9 (118.7 to 123.1)			119.2 (117.3 to 121.0)	
		Crude mean difference (95% CI)		Reference			−1.5 (−3.6 to 0.6)			−1.7 (−4.0 to 0.6)			−*3.2* (−*5.4 to* −*1.0*)	
		Adjusted mean difference (95% CI)		Reference			−1.5 (−3.6 to 0.6)			−1.7 (−4.0 to 0.5)			−*3.2* (−*5.4 to* −*1.0*)	
	Comparison of the adjusted mean differences between the 2 groups (95% CI); *P* value		N/A			−0.7 (−4.8 to 3.4); *P*=.74			4.4 (−0.1 to 8.9); *P*=.05			3.0 (−1.3 to 7.3); *P*=.17		

^a^FBG: fasting blood glucose.

^b^The adjusted model was adjusted for age, sex, and baseline FBG.

^c^Values in italic indicate a significant difference (*P*<.05).

^d^N/A: not applicable.

**Table 4:** Comparison of the postprandial blood glucose levels by diabetes status between the participants in the cohort using only the digital health app and those using the hospital-linked digital health app.

Participants				0 weeks		4 weeks		8 weeks		12 weeks
**Patients without diabetes (normal + prediabetes)**										
	**Digital health app only**									
		PPG^a^ (mg/dL), mean (95% CI)	130.7 (116.8 to 138.6)		135.8 (117.7 to 148.0)		136.8 (116.2 to 151.3)		129.4 (115.9 to 137.0)	
		Crude mean difference (95% CI)	Reference		1.0 (−8.5 to 10.5)		1.9 (−11.6 to 15.4)		−1.2 (−10.3 to 7.8)	
		Adjusted mean difference^b^ (95% CI)	Reference		0.4 (−10.5 to 11.3)		1.8 (−9.7 to 13.4)		−0.4 (−11.8 to 11.0)	
	**Hospital-linked digital health app**									
		PPG (mg/dL), mean (95% CI)	131.0 (125.0 to 137.1)		125.8 (120.4 to 131.3)		126.6 (121.3 to 132.0)		129.6 (122.1 to 137.2)	
		Crude mean difference (95% CI)	Reference		−*6.0* (−*11.8 to* −*0.2*)^c^		−4.2 (−9.7 to 1.3)		−1.4 (−8.7 to 5.9)	
		Adjusted mean difference (95% CI)	Reference		−*5.8* (−*11.4 to* −*0.3*)		−4.2 (−9.8 to 1.4)		−1.7 (−8.3 to 4.9)	
	Comparison of the adjusted mean differences between the 2 groups (95% CI); *P* value			N/A^d^		−6.2 (−18.4 to 6.0); *P*=.31		−6.0 (−18.8 to 6.8); *P*=.35		−1.3 (−14.5 to 11.9); *P*=.84
**Patients with diabetes**										
	**Digital health app only**									
		PPG (mg/dL), mean (95% CI)	162.7 (150.3 to 175.2)		164.6 (152.9 to 176.2)		160.5 (149.9 to 171.1)		170.0 (157.8 to 182.3)	
		Crude mean difference (95% CI)	Reference		3.4 (−7.2 to 13.9)		−2.9 (−13.4 to 7.6)		7.3 (−5.8 to 20.4)	
		Adjusted mean difference (95% CI)	Reference		3.1 (−7.2 to 13.4)		−2.6 (−14.2 to 9.1)		7.3 (−3.9 to 18.5)	
	**Hospital-linked digital health app**									
		PPG (mg/dL), mean (95% CI)	163.1 (156.6 to 169.5)		151.6 (146.3 to 157.0)		151.6 (145.6 to 157.1)		152.1 (146.9 to 157.4)	
		Crude mean difference (95% CI)	Reference		−*11.0* (−*16.9 to* −*5.0*)		−*9.6* (−*16.2 to* −*3.0*)		−*10.9* (−*17.1 to* −*4.7*)	
		Adjusted mean difference (95% CI)	Reference		−*10.9* (−*16.8 to* −*5.0*)		−*9.7* (−*16.0 to* −*3.3*)		−*10.9* (−*17.4 to* −*4.7*)	
	Comparison of the adjusted mean differences between the 2 groups (95% CI); *P* value			N/A		−*14.0* (−*25.9 to* −*2.1*)*;* *P=.02*		−7.1 (−20.4 to 6.2); *P*=.29		−*18.2* (−*31.1 to* −*5.3*)*;* *P=.01*

^a^PPG: postprandial blood glucose.

^b^The adjusted model was adjusted for age, sex, and baseline PPG.

^c^Values in italic indicate a significant difference (*P*<.05).

^d^N/A: not applicable.

## Discussion

### Main Findings and Plausible Mechanism

We observed that the participants with diabetes using the hospital-linked digital health app exhibited a lower PPG after 12 weeks compared with those with diabetes using only the digital health app. After 12 weeks, a hospital-linked digital health app was found to reduce SBP and DBP in participants without hypertension and FBG levels in all participants; however, there was no statistically significant difference compared to the control group (using only digital health app).

The Asian diet, particularly the Korean diet, is typically characterized by high carbohydrate intake. Furthermore, consuming high amounts of carbohydrates has been linked to increased risks of metabolic syndrome and type 2 diabetes [1-3], affecting insulin secretion and PPG levels. Nevertheless, our results indicated no significant differences were observed between dietary glycemic load and FBG. Previous research has also described that PPG is an independent risk factor for mortality in newly diagnosed patients with type 2 diabetes, while FBG is not [5]. Lowering PPG is crucial for controlling overall glucose levels and improving long-term outcomes [1-3]. Additionally, the difference in FBG before and after an intervention is smaller than that of PPG, further emphasizing the importance of lowering PPG in diabetes therapy. This study indicates that the hospital-linked digital health app can decrease PPG but not FBG in patients with diabetes.

This may be a result of the use of a more comprehensive application that enables users to consult telemedicine physicians through BP and glucose measures; medication and exercise; notifications of upcoming doctor visits; records of BP and glucose control; and drugs, training modules, and health assessments [20]. Between scheduled visits to the hospital, automatic integration of glucose and BP results and health diary data in the electronic medical records enhances the physician's workflow for data analysis, enhances patient communication, and ultimately results in improved therapy [21]. These findings are expected to play a significant role in accelerating the development and adoption of digital health care devices in the future [22].

### Comparison With Previous Studies

The majority of the research that has examined the BP and glucose-lowering effects of digital health technology interventions in patients with hypertension or diabetes has been published within the last 3 years [23,24]. Recent systematic reviews of studies on hypertension or diabetes that were performed in the United States (n=52 to n=333) [25-27], Canada (n=98) [28], Iceland (n=30) [29], Mexico (n=202) [30], India (n=90) [31], Iran (n=120) [32], China (n=63 to n=480) [33-35], and Japan (n=146 to n=390) [36,37], as well as 2 studies performed in South Korea (n=148 and n=182) [38,39], were included in the comparison with our study. The only previous studies that are currently available are of moderate quality, and the lack of blinding and limited sample size are the main problems that eventually cause bias [23]. Few studies have been conducted on mobile health apps that can control diabetes and hypertension concurrently [23]. Most of the studies on BP and glucose changes were often measured through prescription records recorded by clinicians.

However, this study evaluated changes in BP and glucose using mobile health apps in more than 2000 participants in South Korea. Additionally, there were divided into systolic/diastolic, and blood glucose levels were separated into pre-and postprandial statuses. The SE of the mean was minimized in this study by measuring BP and glucose levels using electronic medical records while not seeing a doctor.

### Limitations

This study has several limitations. First, although previous research has suggested that diabetes and hypertension may have distinct impacts such as individuals’ age, this study did not investigate these conditions by dividing the participants into age-related subgroups [40]. Therefore, the findings for digital health technology on BP and glucose may be restricted to determining whether they are particular issues with digital treatments. In further studies, a larger number of participants, which is possible to categorize them into age-related subgroups, would allow for a more accurate impact of digital health technology on BP and glucose levels excluding age-related factors. Second, as multimorbidity is increasing due to the aging population, it is important to consider the claim that individuals with comorbidities benefited less from the intervention. In further studies, dividing the participants into age-related subgroups could potentially help adjust for multimorbidity based on age. Third, some people may not have access to or be able to use a smartphone or digital therapy apps if they have a lower degree of technology literacy [41]. To prevent a lack of enthusiasm and a rejection of the value of telemedicine due to the low skill level of information and communications technology, education on the use and implementation of digital treatment is required. Fourth, the findings might not be generalizable to other regions of the world, because our data only included South Korean participants. Fifth, the number of participants was still small even if the sample size was larger than in the previous study. International randomized controlled trials with large sample sizes are required to examine the effects of digital health apps that support BP and glucose monitoring.

### Clinical Implications

At least one-third of patients are self-monitoring, and the majority of general practitioners use it to control hypertension or diabetes [42]. Digital health technologies were successfully used to help with contact tracking, isolation management, primary care improvement, and communication between citizens and decision-makers during the COVID-19 epidemic [43,44]. These interventions have been shown to have several clinical advantages; therefore, it is critical to learn how to remotely control BP and glucose [42]. Previous research demonstrated that digital health technology can positively impact cost-effectiveness in terms of both costs and health outcomes [45,46]. Particularly, using new mobile apps or website interventions, the data demonstrated a favorable impact [45,47]. The factors impacting BP and glucose should be taken into account for the successful implementation of such interventions, and it would be crucial to communicate the objectives and advantages set by health care professionals and patients using as much user feedback as feasible [45,48]. Additionally, it is crucial to research and record digital therapies that can successfully lower target BP and glucose levels [23]. Access to technologies like smartphones and the internet, as well as the difficulties of not being fully integrated into the current health care systems, must be addressed in terms of cost and logistics [45,49]. These challenges could potentially be addressed through the development of hospital-linked digital health technology.

### Conclusions

Hospital-linked digital interventions have greatly improved glucose control for diabetes compared with using digital health technology only. These hospital-linked digital health apps have the potential to offer consumers and health care professionals cost-effective support in decreasing glucose levels when used in conjunction with self-monitoring.

## Appendix

Multimedia Appendix 1 The process of using the WellCheck app and web.

Multimedia Appendix 2 Density and box plot of propensity scores before and after matching in cohort A, B, and C.

## Abbreviations

BP: blood pressure
DBP: diastolic blood pressure
FBG: fasting blood glucose
PPG: postprandial glucose
SBP: systolic blood pressure

## Data Availability

The data set supporting the results of our study is available from the corresponding author upon reasonable request, who uses the full data set and takes responsibility for its integrity and analysis.
