# Supplementary material for: Real-World Evidence of a Hospital-Linked Digital Health App for the Control of Hypertension and Diabetes Mellitus in South Korea: Nationwide Multicenter Study
Source: JMIR Form Res. 2023 Aug 21;7:e48332. doi: 10.2196/48332 (PMC10477930; doi:10.2196/48332)
Supplement: Multimedia Appendix 1 [file formative_v7i1e48332_app1.pdf]

Well Check

The process of using Well-check App & Web

User App process

- Blood pressure
- Blood sugar
- Medication
- Etc. (Message from the doctor / Health infomation)

Doctor Web process

- Blood pressure
- Blood sugar
- Medication
- Etc. (Message from the doctor / Monitoring)

2023. 03. 14

01 (App)

Blood pressure

The process of using Well-check_App

- 1. Tutorial for measuring blood pressure
- 2. How to input blood pressure
- 3. Feedback on blood pressure
- 4. View blood pressure records

[The process of using Well-check] blood pressure_App

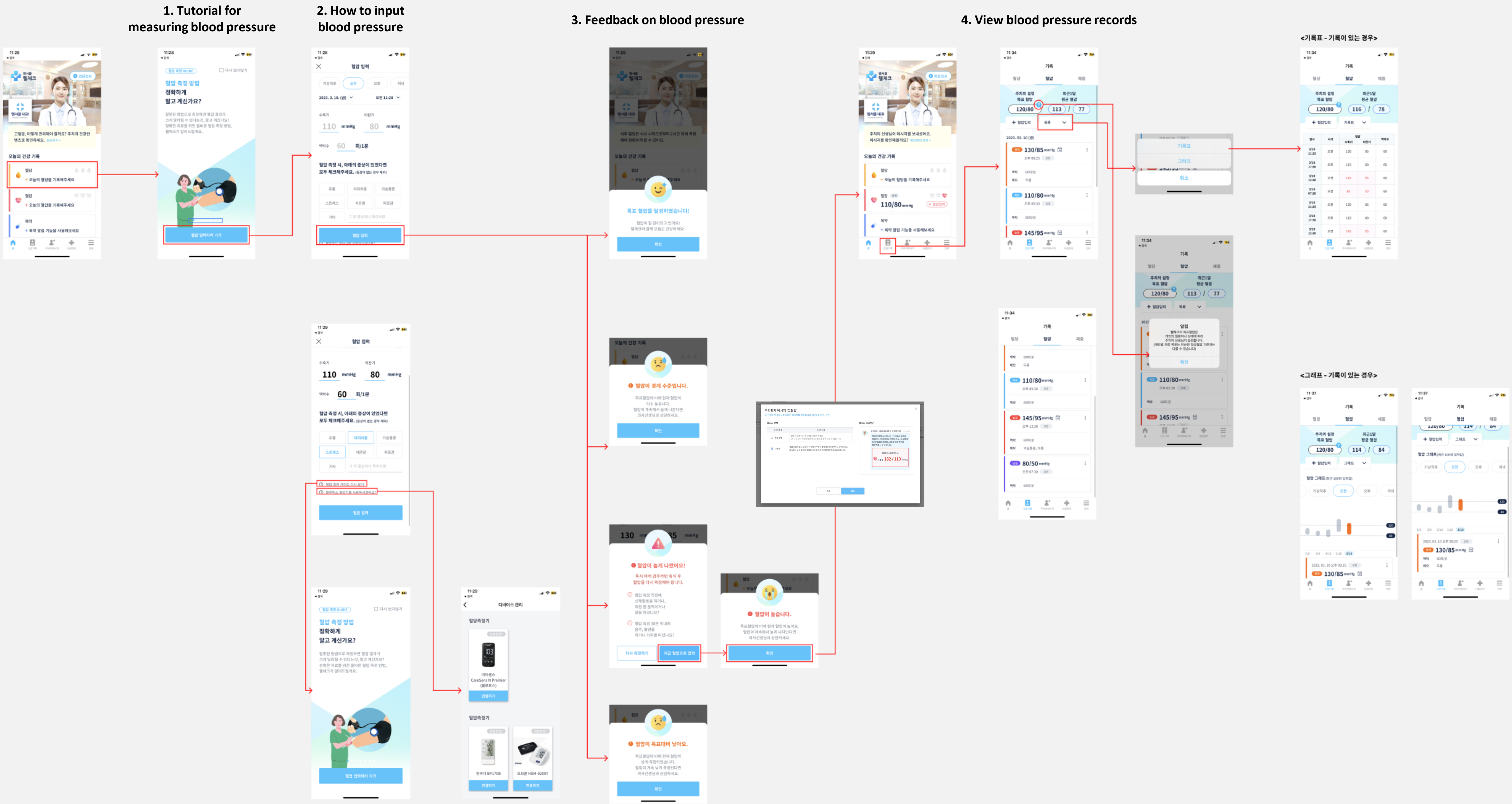

[The process of using Well-check] Blood pressure_App

1. Tutorial for measuring blood pressure

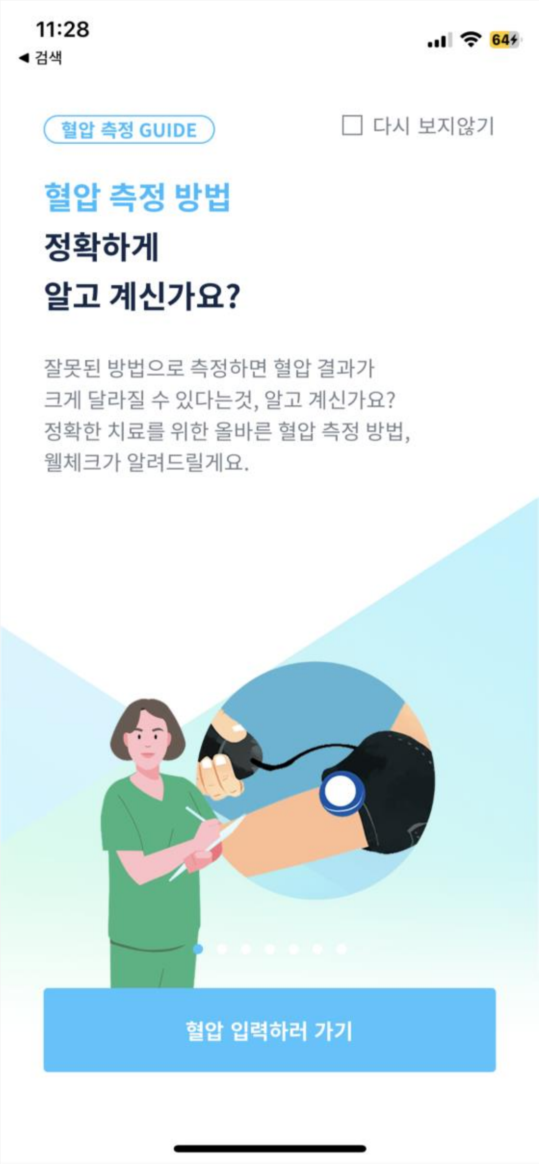

Based on the guidelines of the Diabetes and Hypertension Society, Well-check provides step-by-step illustrations to help patients easily and accurately understand the blood pressure measurement process.

[The process of using Well-check] Blood pressure_App

2. How to input blood pressure

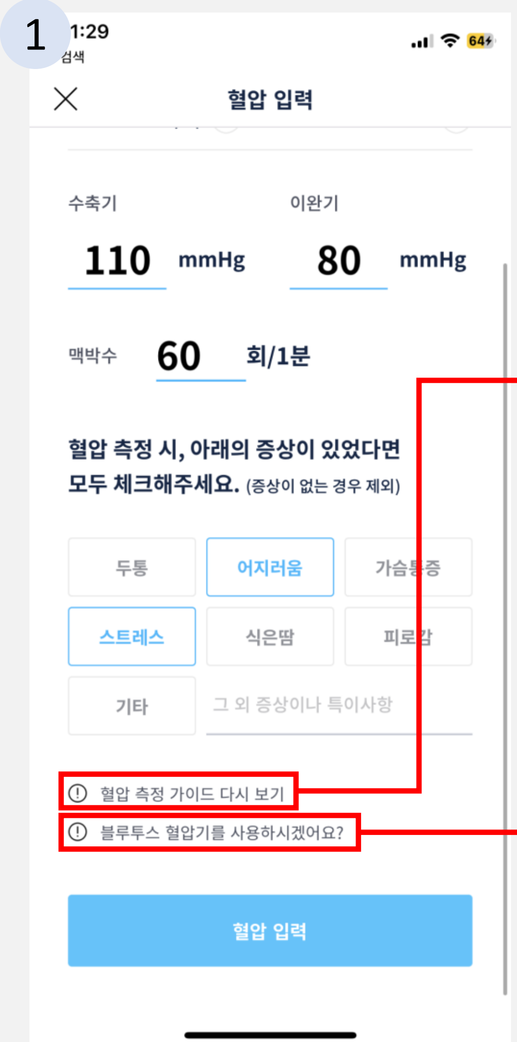

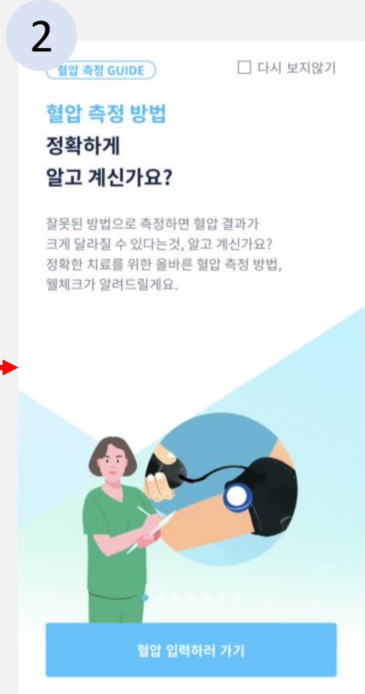

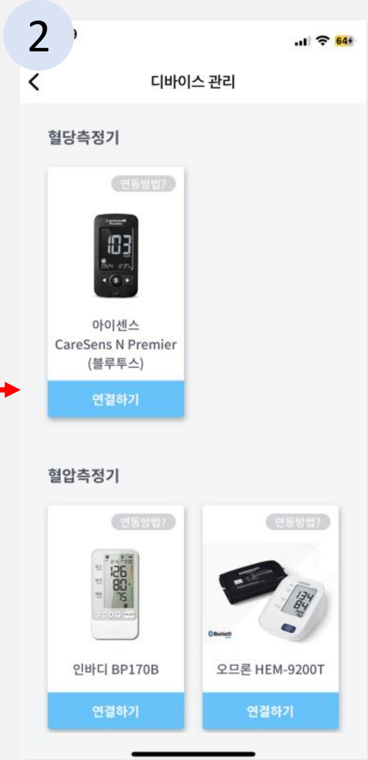

1

Rather than simply recording the numbers, we encourage patients to also record meaningful symptoms to help their doctor accurately assess the situation and determine whether medical intervention is necessary. This will help the doctor accurately assess why blood pressure is increasing.

2

The blood pressure measurement guide can be reviewed at any time, and if you connect a Bluetooth blood pressure monitor, you can use it conveniently.

[The process of using Well-check] Blood pressure_App

3. Feedback on blood pressure

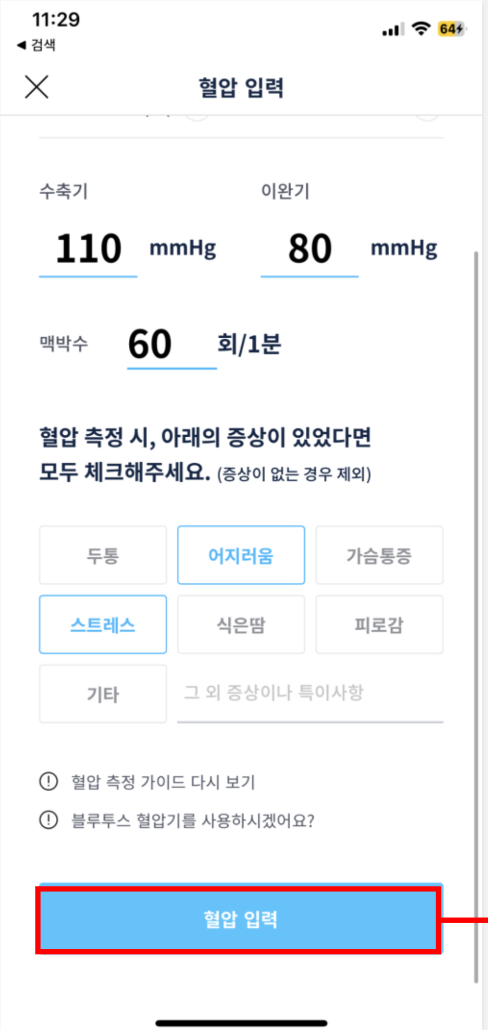

When patients enter their blood pressure reading, they will receive automatic feedback by comparing their individualized blood pressure and blood sugar targets set by their doctor based on their hypertension and diabetes diagnosis.

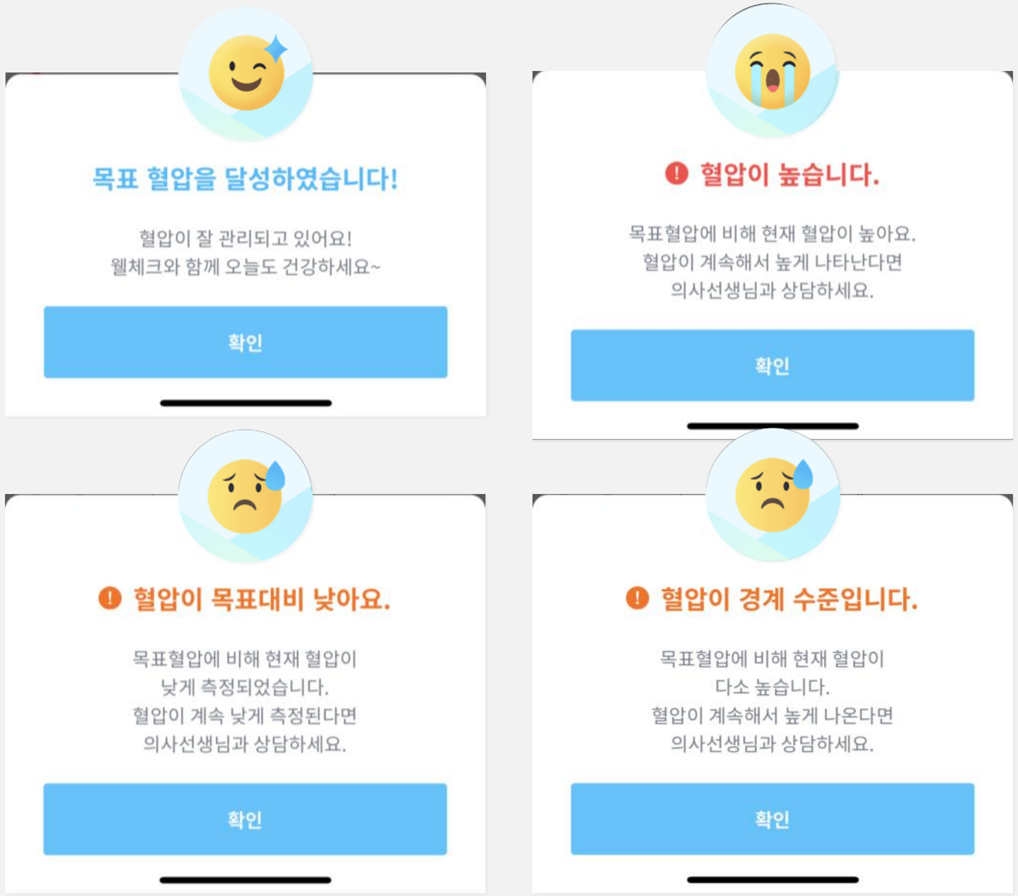

[The process of using Well-check] Blood pressure_App

3. Feedback on blood pressure (Medical intervention)

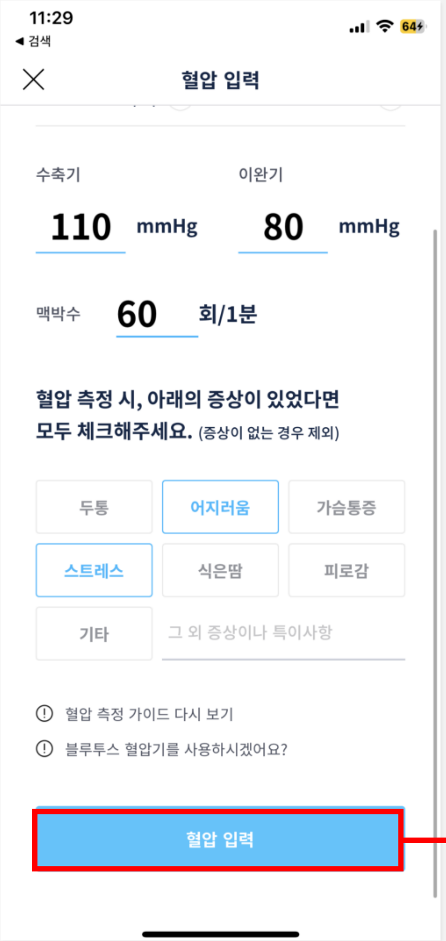

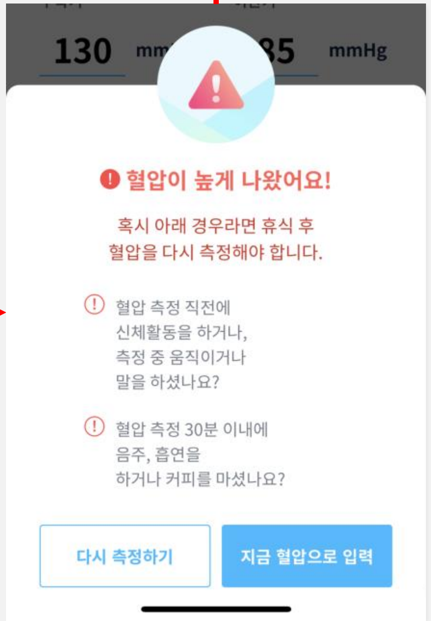

#Feedback through doctor monitoring

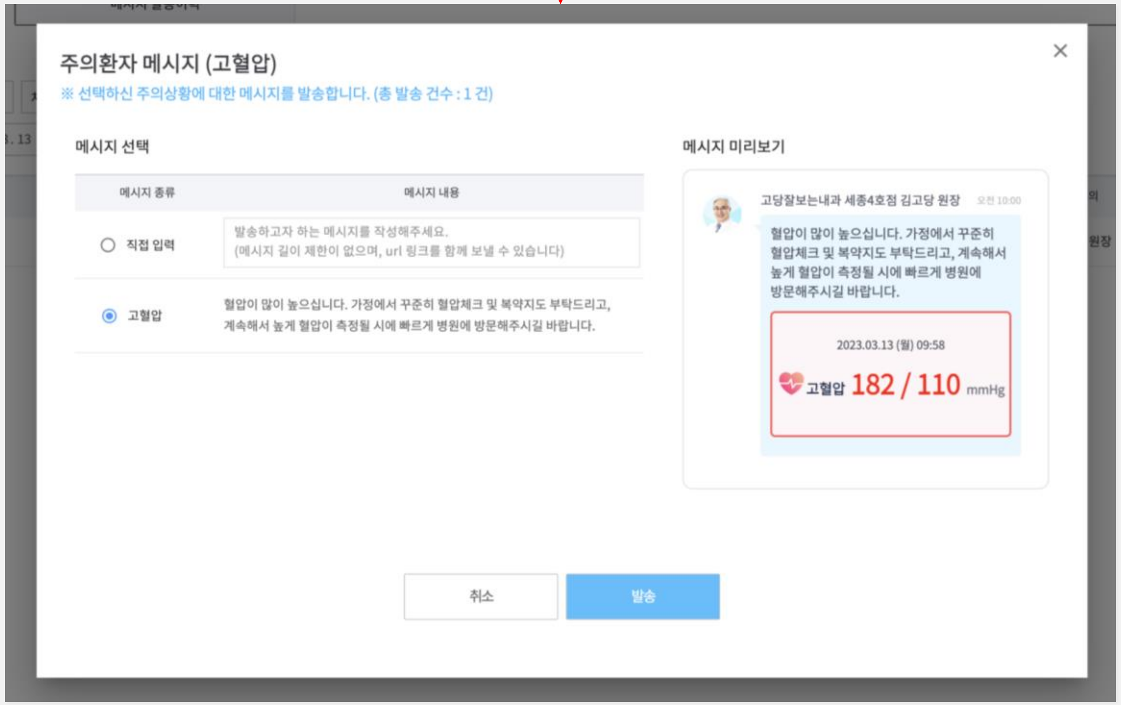

Based on patient’s hypertension and diabetes diagnosis, the doctor sets individualized blood pressure and blood sugar targets for the patient, and patient will receive automatic feedback based on entered readings by comparing them to those targets.

If the entered blood pressure is too high or low compared to the target blood pressure, an alert will be sent to the doctor, and they can send feedback messages to manage it based on their judgment.

[The process of using Well-check] Blood pressure_App

4. View blood pressure records

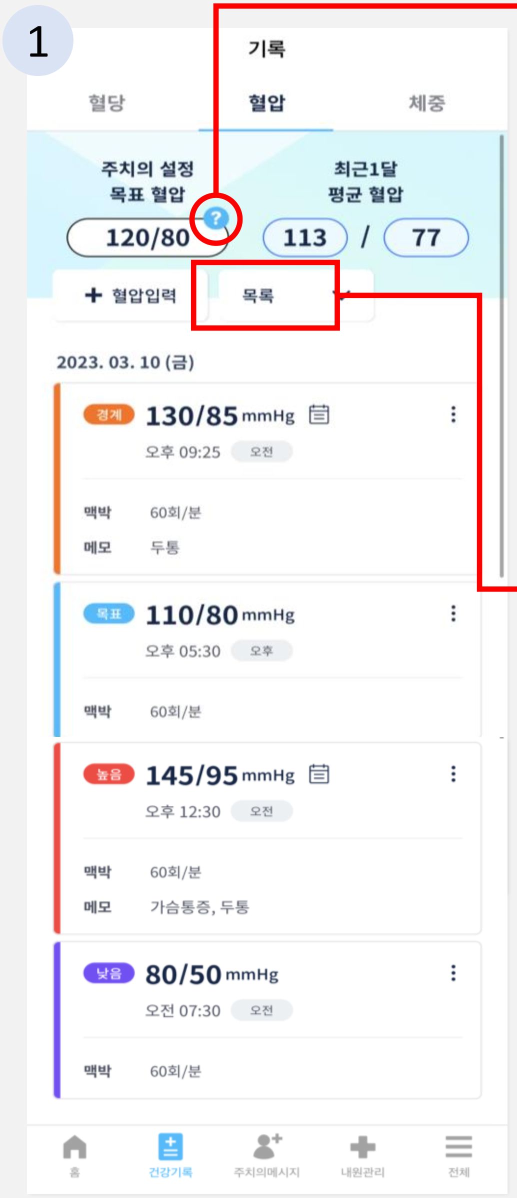

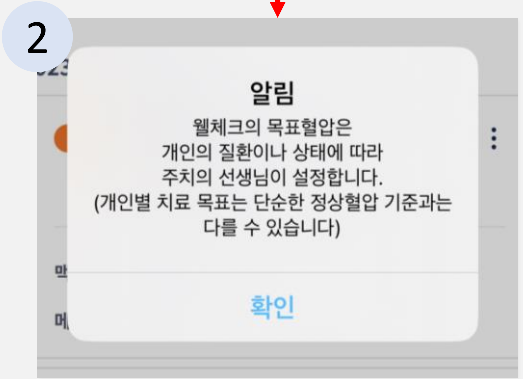

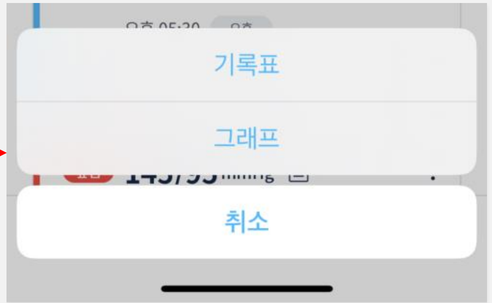

1

In the health record tab, patients can view blood pressure records and evaluate progress by comparing them to your personal blood pressure target, which is color-coded (high: red, low: purple, caution: orange, target: blue).

2

Explanation of the standard for the target blood pressure level.

3

Blood pressure(Blood sugar, weight) records can be viewed in list form, tables, or graph form.

[The process of using Well-check] Blood pressure_App

4. View blood pressure records

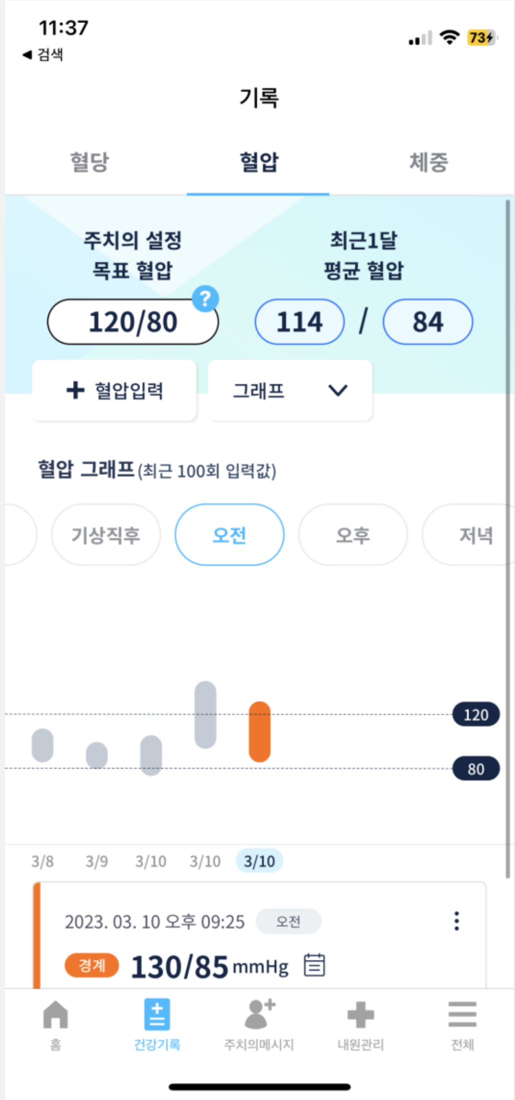

< graph format >

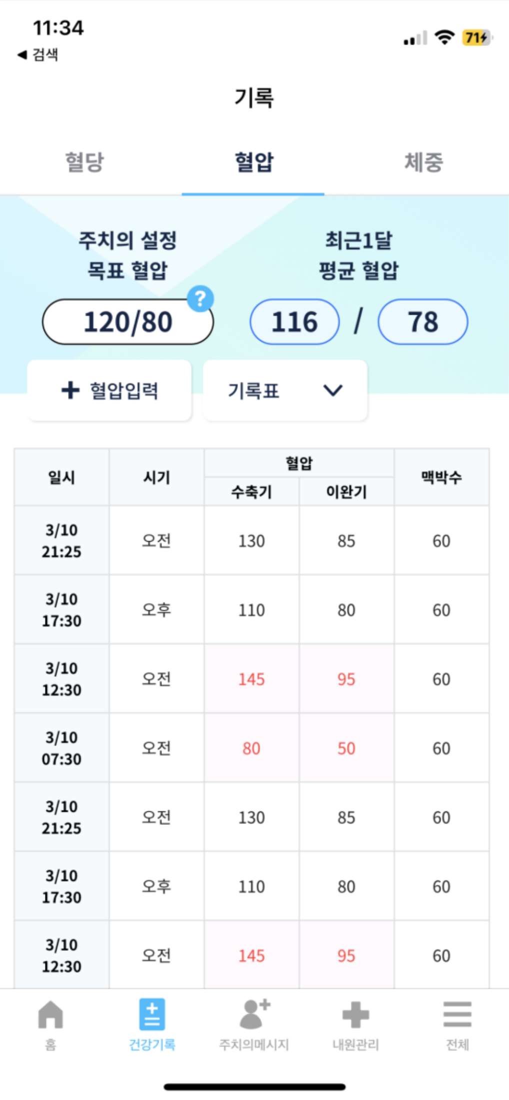

< table format >

The table format is convenient for identifying cases where the blood pressure falls outside the target range. The graph format is useful for understanding the high and low trends of blood pressure.

In addition, it helps motivate patients by showing how well their blood pressure has been managed compared to the target over the past month.

02 (App)

Blood sugar

The process of using Well-check_App

- 1. Tutorial for measuring blood sugar
- 2. How to input blood sugar
- 3. Feedback on blood sugar / Indication of blood glucose recording frequency
- 4. View blood sugar records

[The process of using Well-check] Blood sugar_App

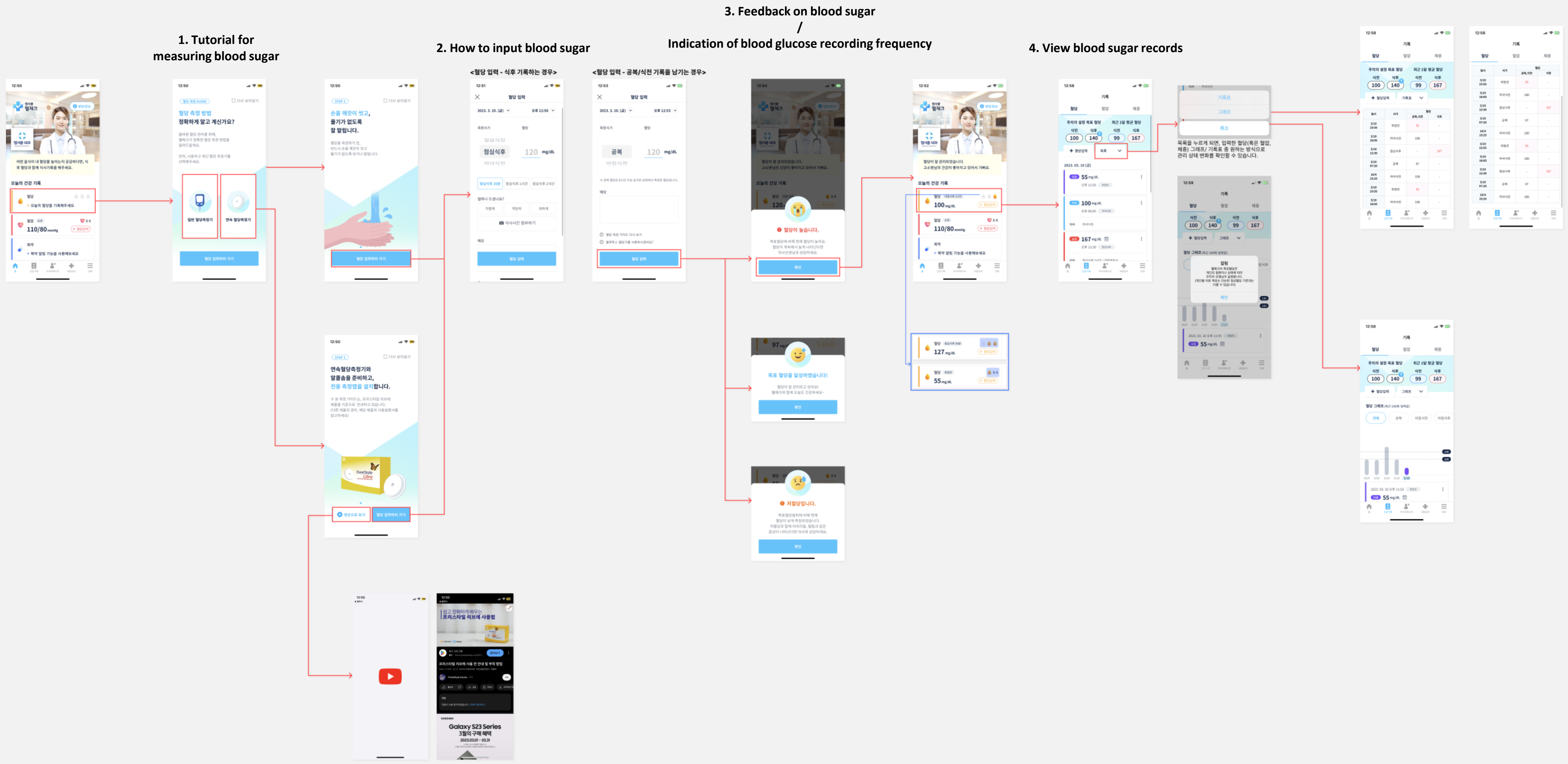

[The process of using Well-check] Blood sugar_App

1. Tutorial for measuring blood sugar

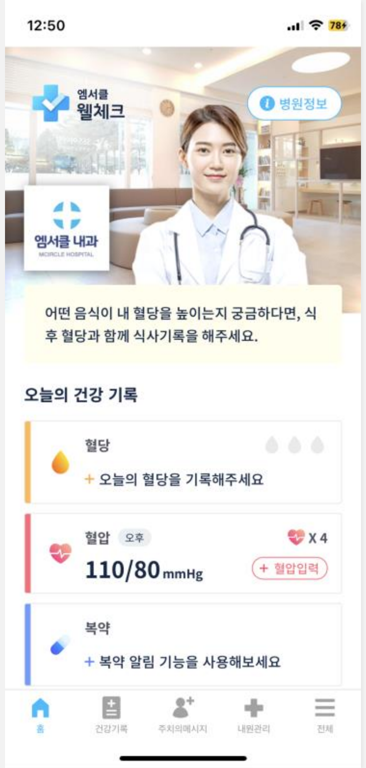

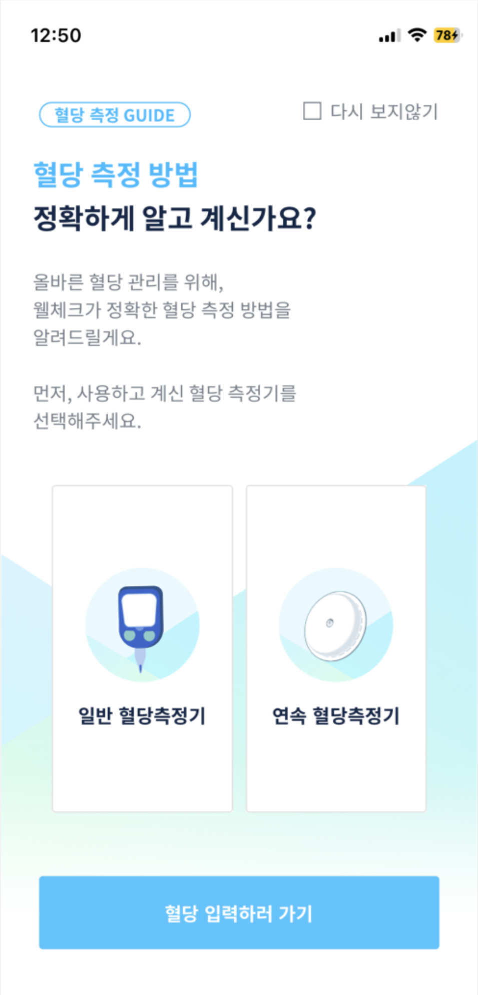

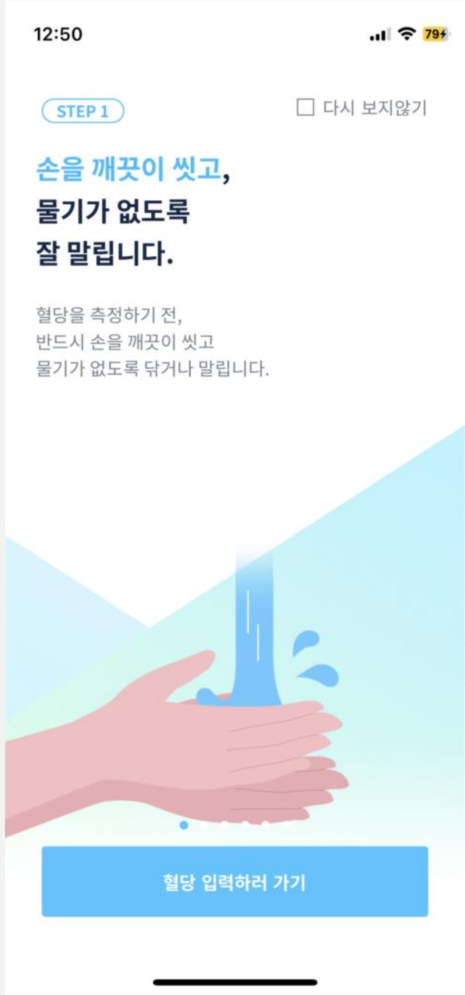

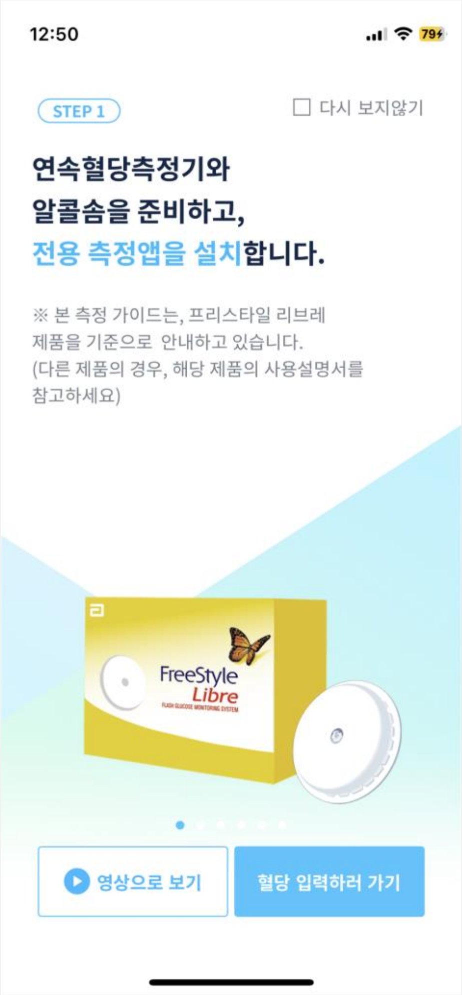

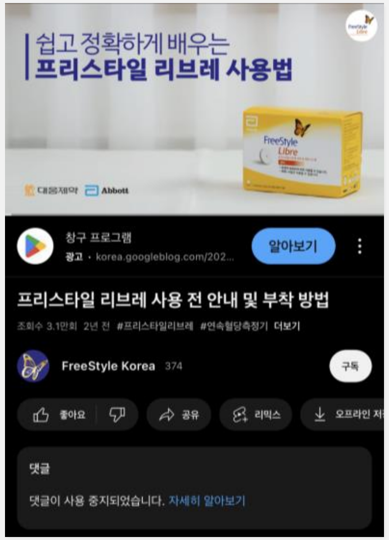

Detailed information on how to use a continuous blood sugar monitor can also be found in video format.

Well-check provides an easy-to-understand guide on how to measure blood sugar using a continuous blood sugar monitor to assist patients in measuring their blood sugar.

[The process of using Well-check] Blood sugar_App

2. How to input blood sugar

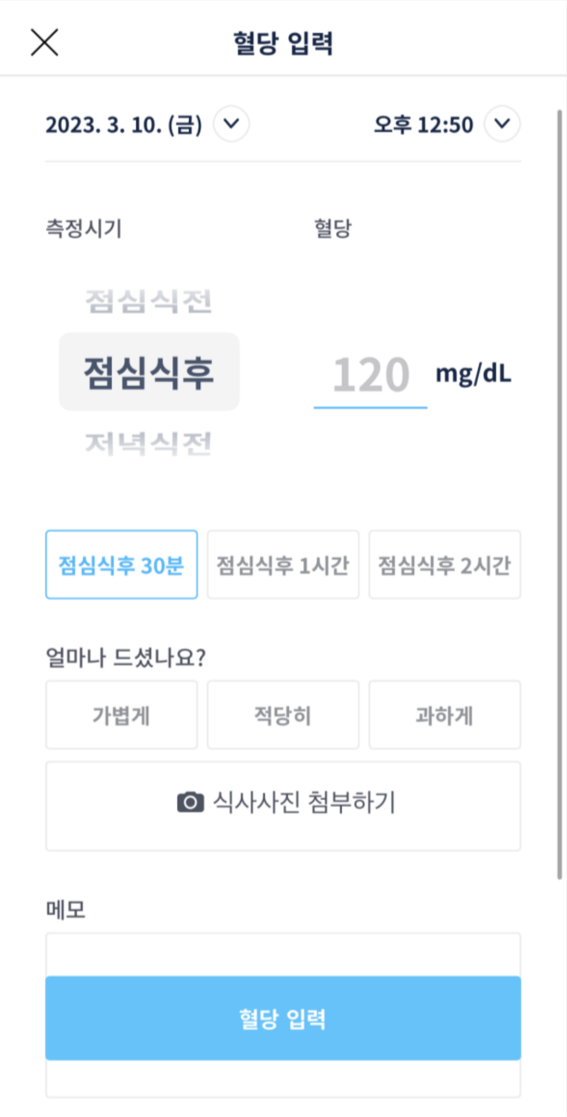

Instead of just recording the numbers, Well-check encourages patients to record significant symptoms along with their blood sugar readings. This allows for a better understanding of why blood sugar levels may be high and whether medical intervention is truly necessary. It also helps the doctor make an accurate assessment of the patient's condition at the time.

[The process of using Well-check] Blood sugar_App

3. Feedback on blood sugar / Indication of blood glucose recording frequency

When patients enter their blood sugar level, depending on whether patients have hypertension or diabetes, they will automatically receive feedback by comparing their personal blood pressure and blood sugar targets set by their doctors.

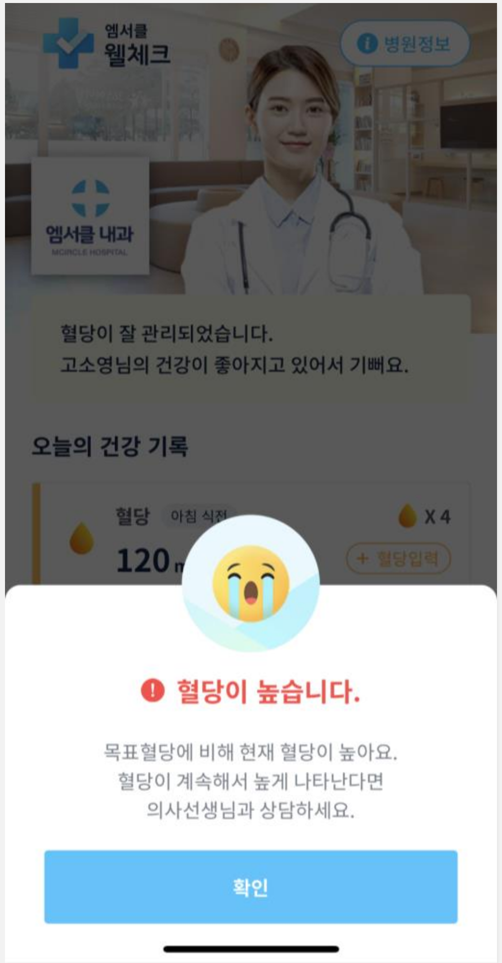

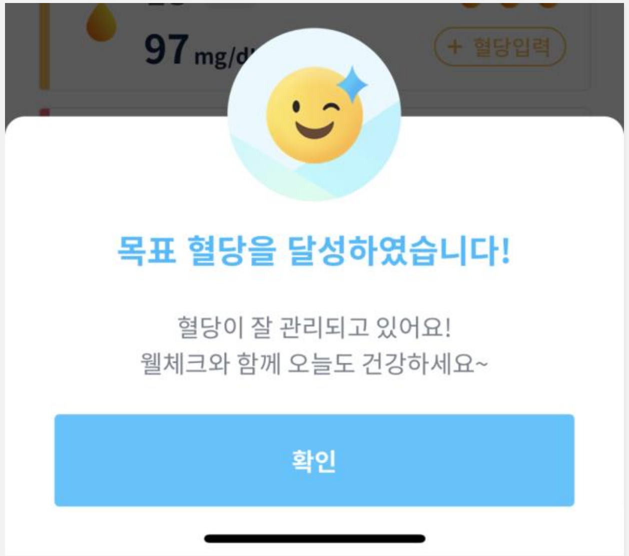

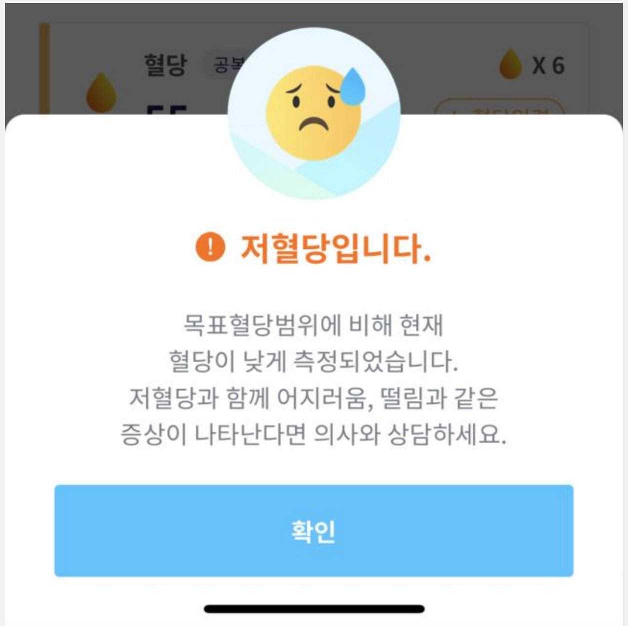

[The process of using Well-check] Blood sugar_App

3. Feedback on blood sugar / Indication of blood glucose recording frequency

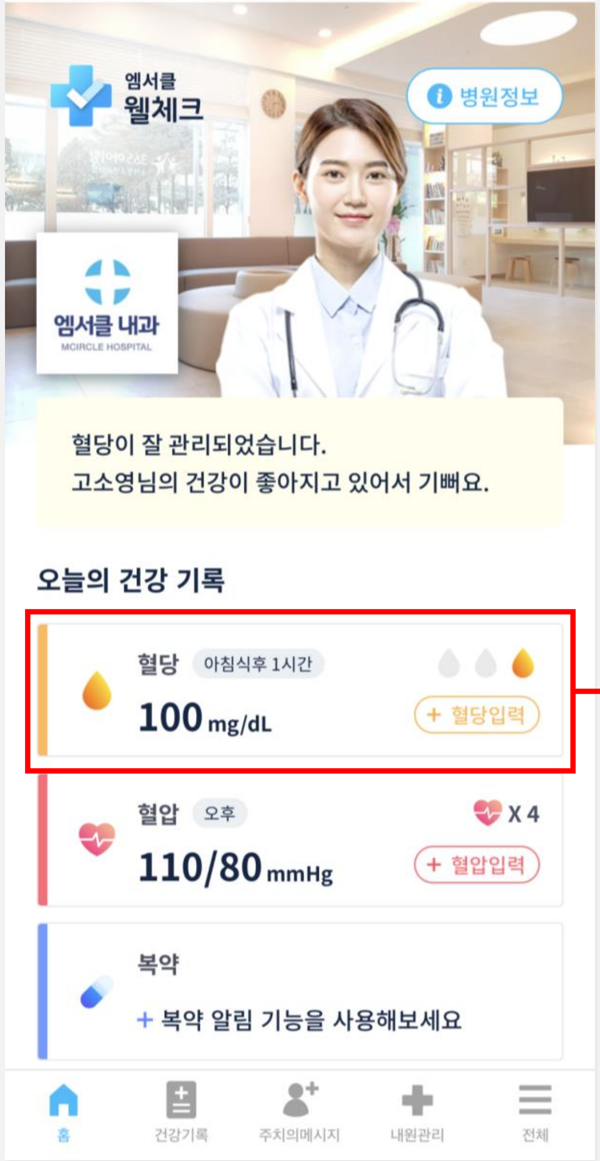

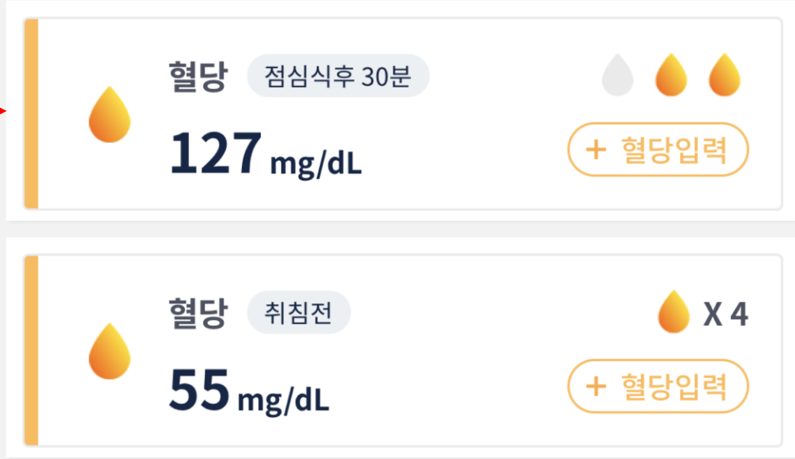

Every time patients enter their blood sugar level, the corresponding icon is colored so that patients can easily see how much blood sugar they have entered on the home screen.

[The process of using Well-check] Blood sugar_App

4. View blood sugar records

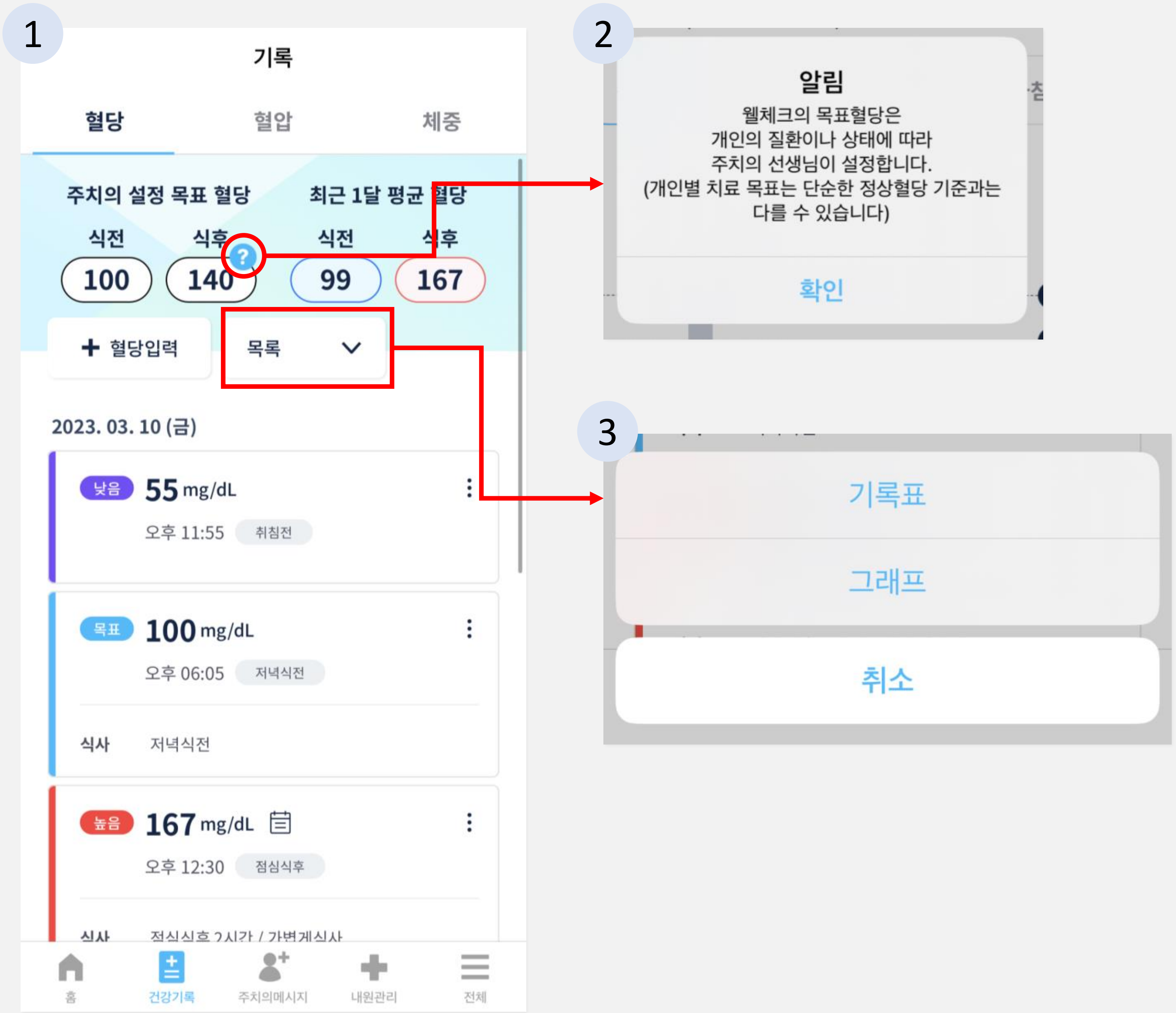

1

In the health record tab, patients can view their blood sugar records and evaluate their progress by comparing them to their personal blood sugar target, which is color-coded (high: red, low: purple, caution: orange, target: blue).

2

Explanation of the standard for the target blood sugar level.

3

Blood sugar (blood pressure, weight) records can be viewed in list form, tables, or graph form.

[The process of using Well-check] Blood sugar_App

4. View blood sugar records

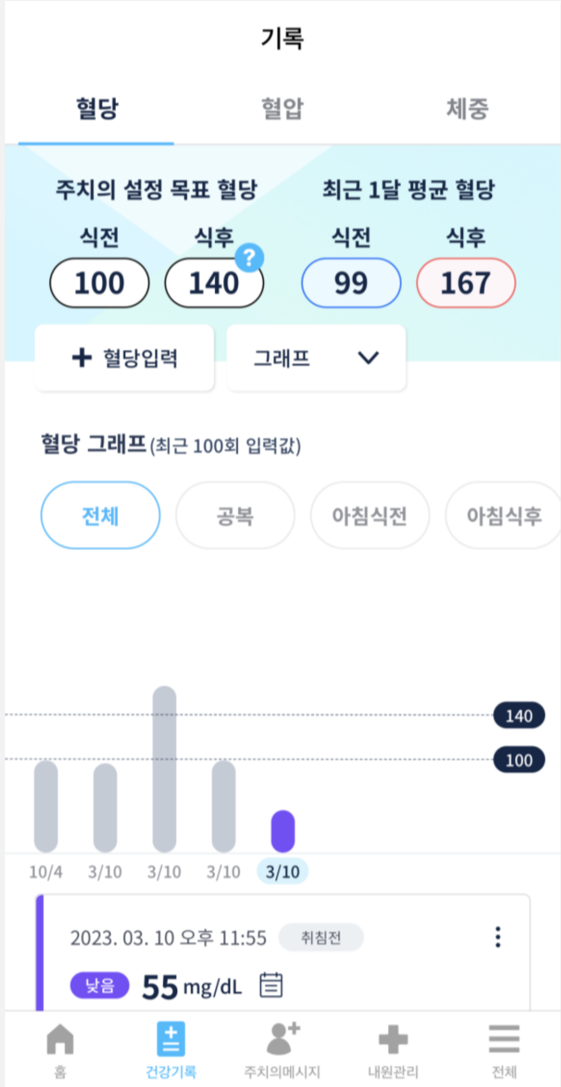

<보기-그래프>

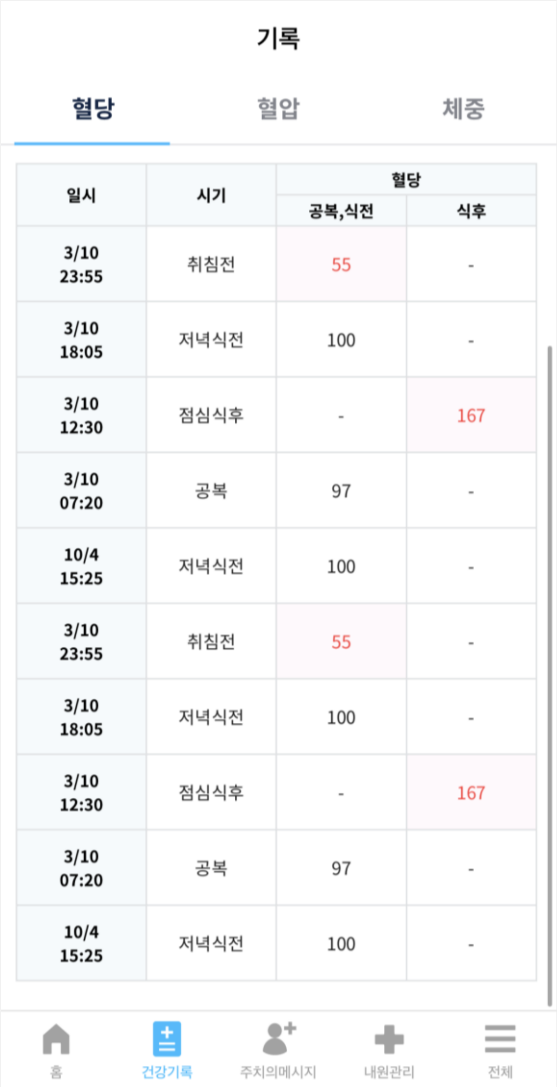

일시	시기	혈당	
		공복,식전	식후
3/10 23:55	취침전	55	-
3/10 18:05	저녁식전	100	-
3/10 12:30	점심식후	-	167
3/10 07:20	공복	97	-
10/4 15:25	저녁식전	100	-
3/10 23:55	취침전	55	-
3/10 18:05	저녁식전	100	-
3/10 12:30	점심식후	-	167
3/10 07:20	공복	97	-
10/4 15:25	저녁식전	100	-

<보기-기록표>

The table format is convenient for identifying cases where the blood sugar falls outside the target range. The graph format is useful for understanding the high and low trends of blood sugar.

In addition, it helps motivate patients by showing how well their blood sugar has been managed compared to the target over the past month.

03 (App)

Medication

The process of using Well-check_App

- 1. Add medication reminder / Check and record medication reminders
- 2. Enter medication record
- 3. Record a prescription (Upload a photo)

[The process of using Well-check] Medication_App

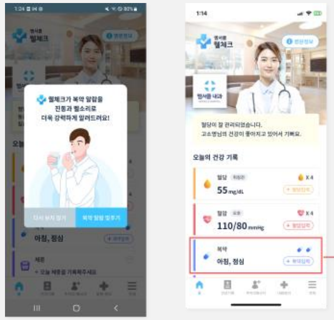

1. . Add medication reminder / Check and record medication reminders

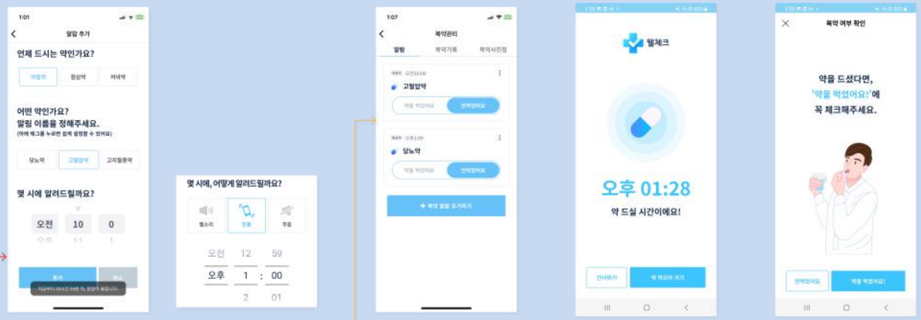

2. Enter medication record

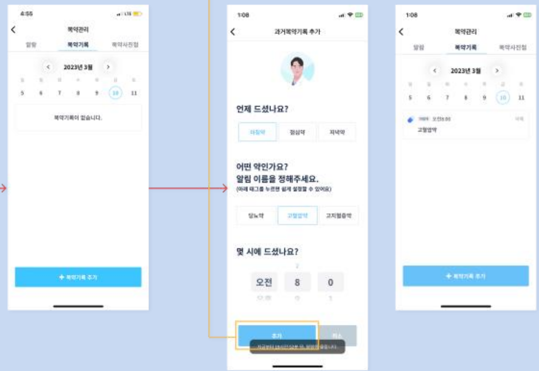

3. Record a prescription (Upload a photo)

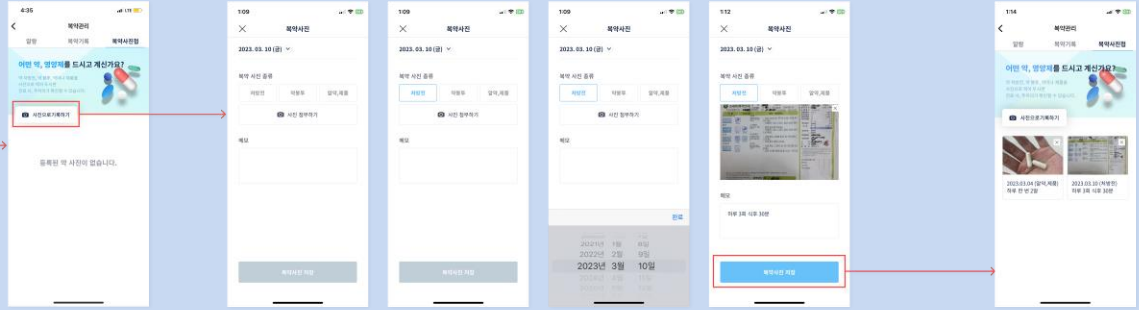

[The process of using Well-check] Medication_App

1. Add medication reminder / Check and record medication reminders

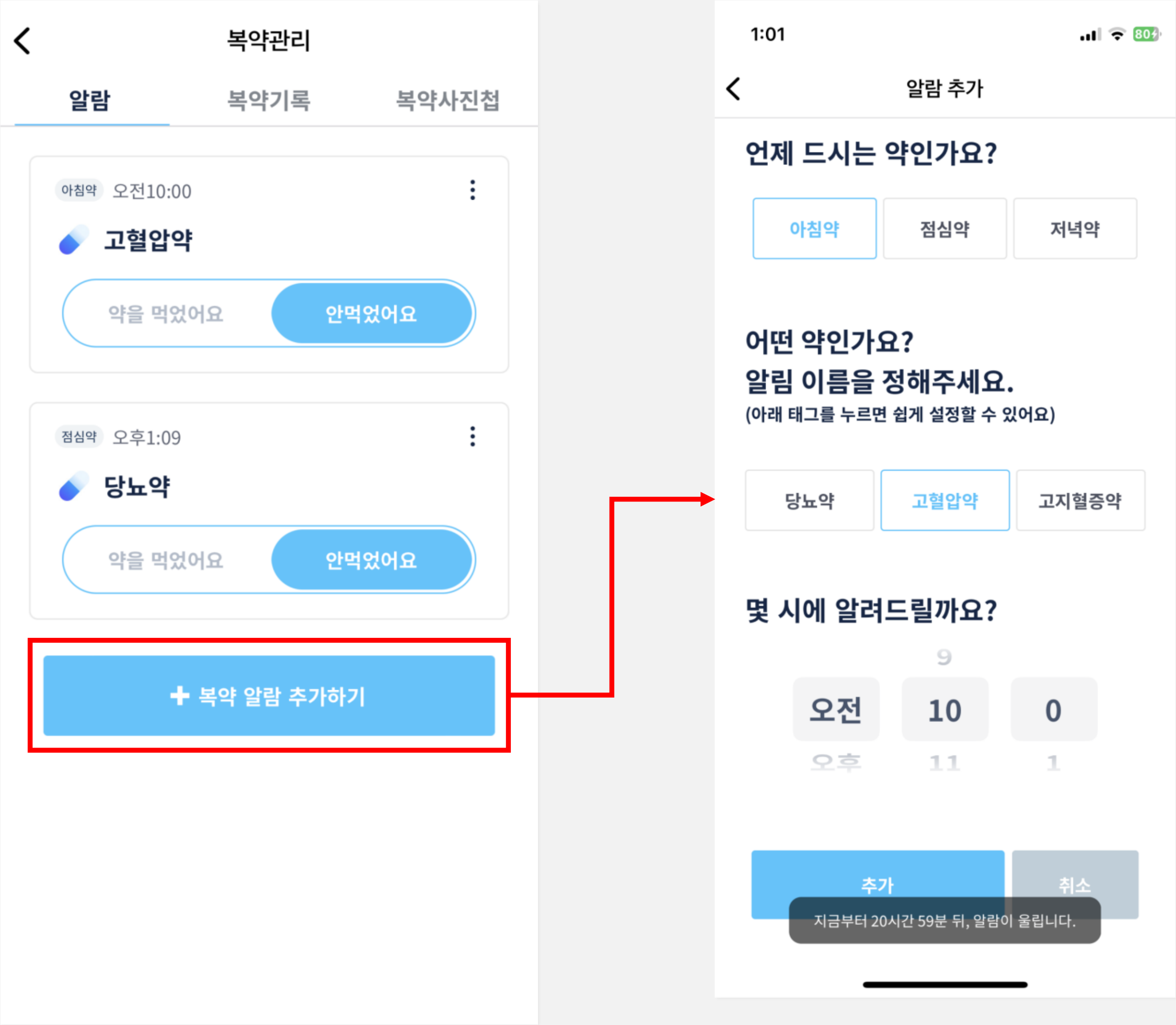

Patients can check whether they have taken their medication or not and add a medication reminder if needed.

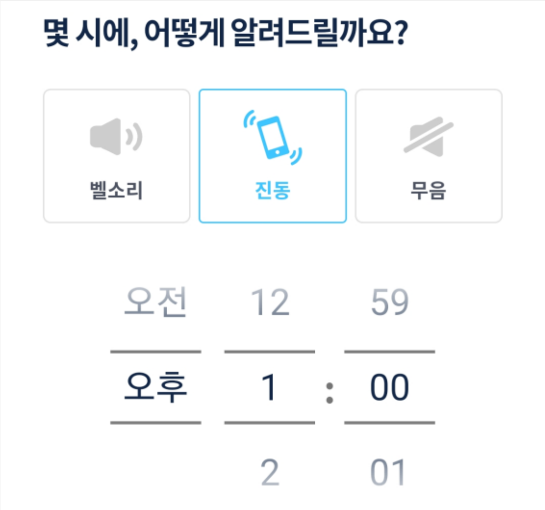

[The process of using Well-check] Medication_App

1. Add medication reminder / Check and record medication reminders

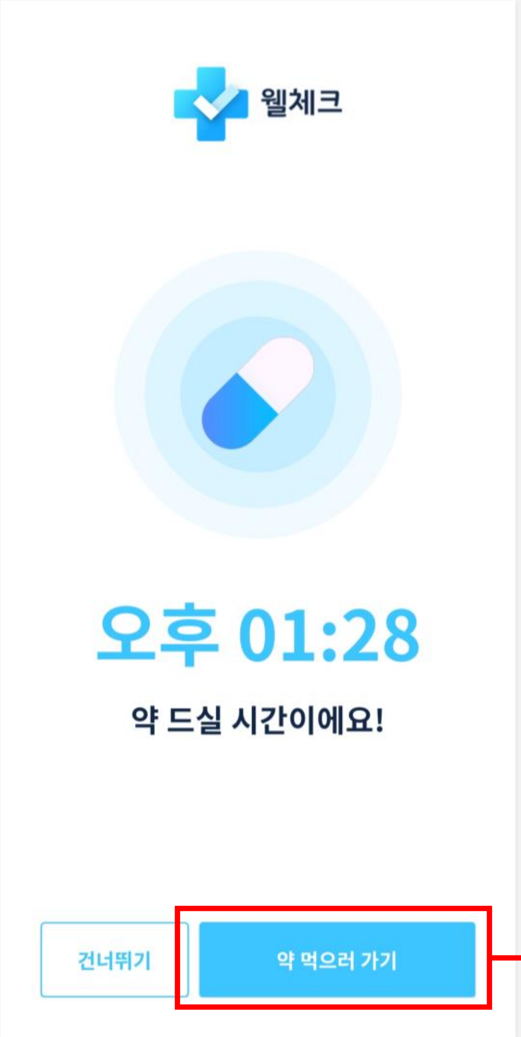

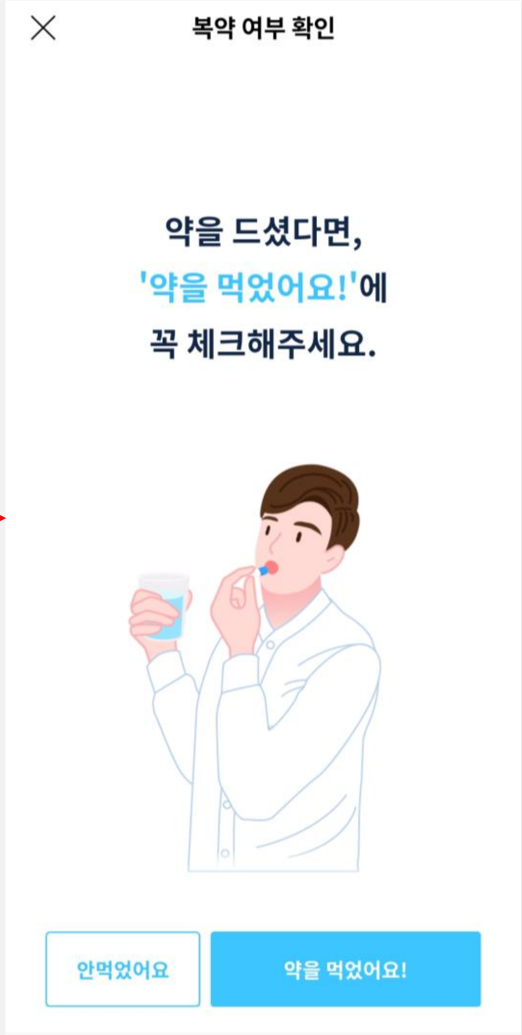

When turning off the alarm, patients can check whether they have taken their medication or not. Based on this, the doctor can also verify the patient's medication adherence.

[The process of using Well-check] Medication_App

2. Enter medication record

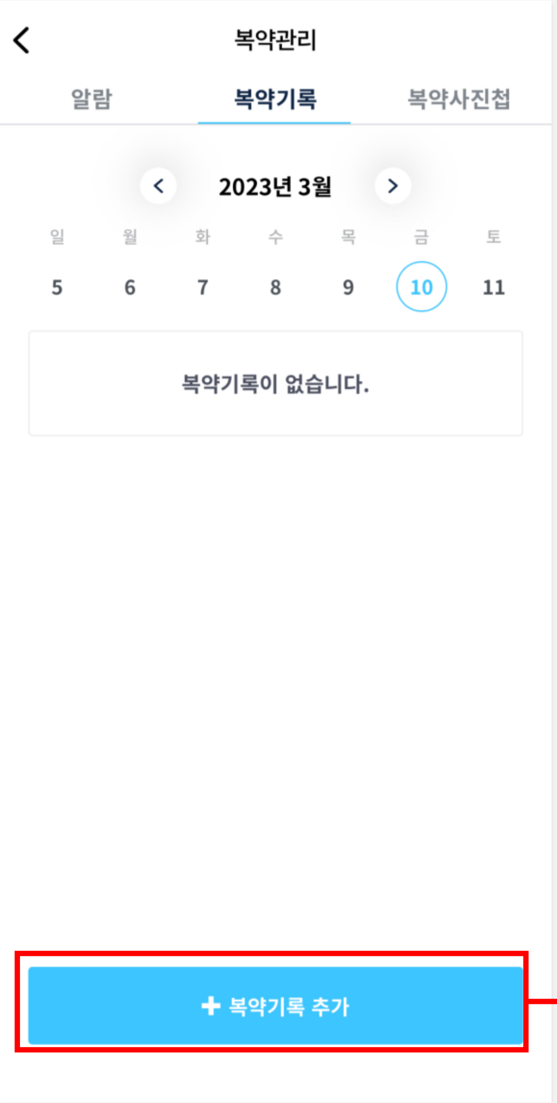

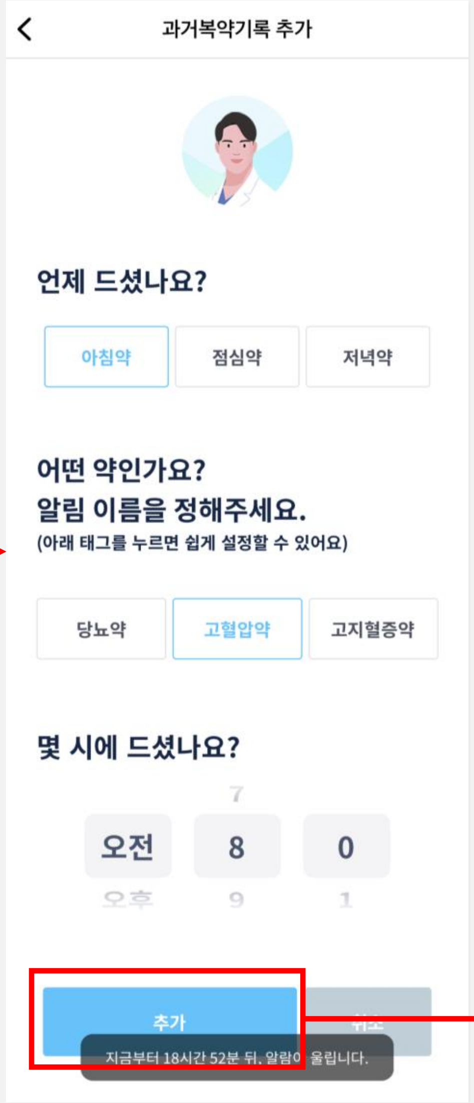

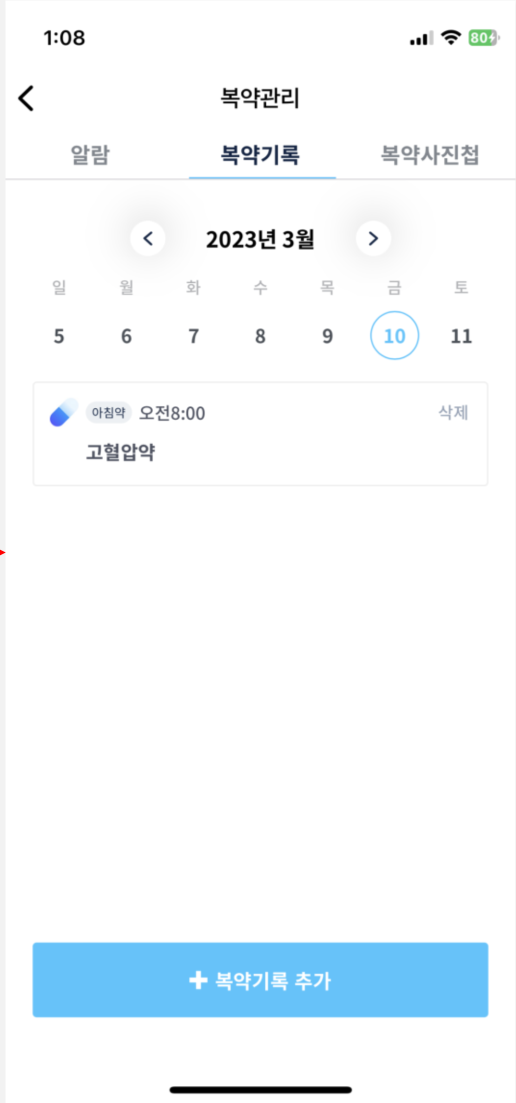

Patients can add medication records in the medication record tab.

When recording medication, a notification is automatically set up to help users with chronic diseases who need to continuously take medication remember and consistently take medication in the future.

[The process of using Well-check] Medication_App

3. Record a prescription (Upload a photo)

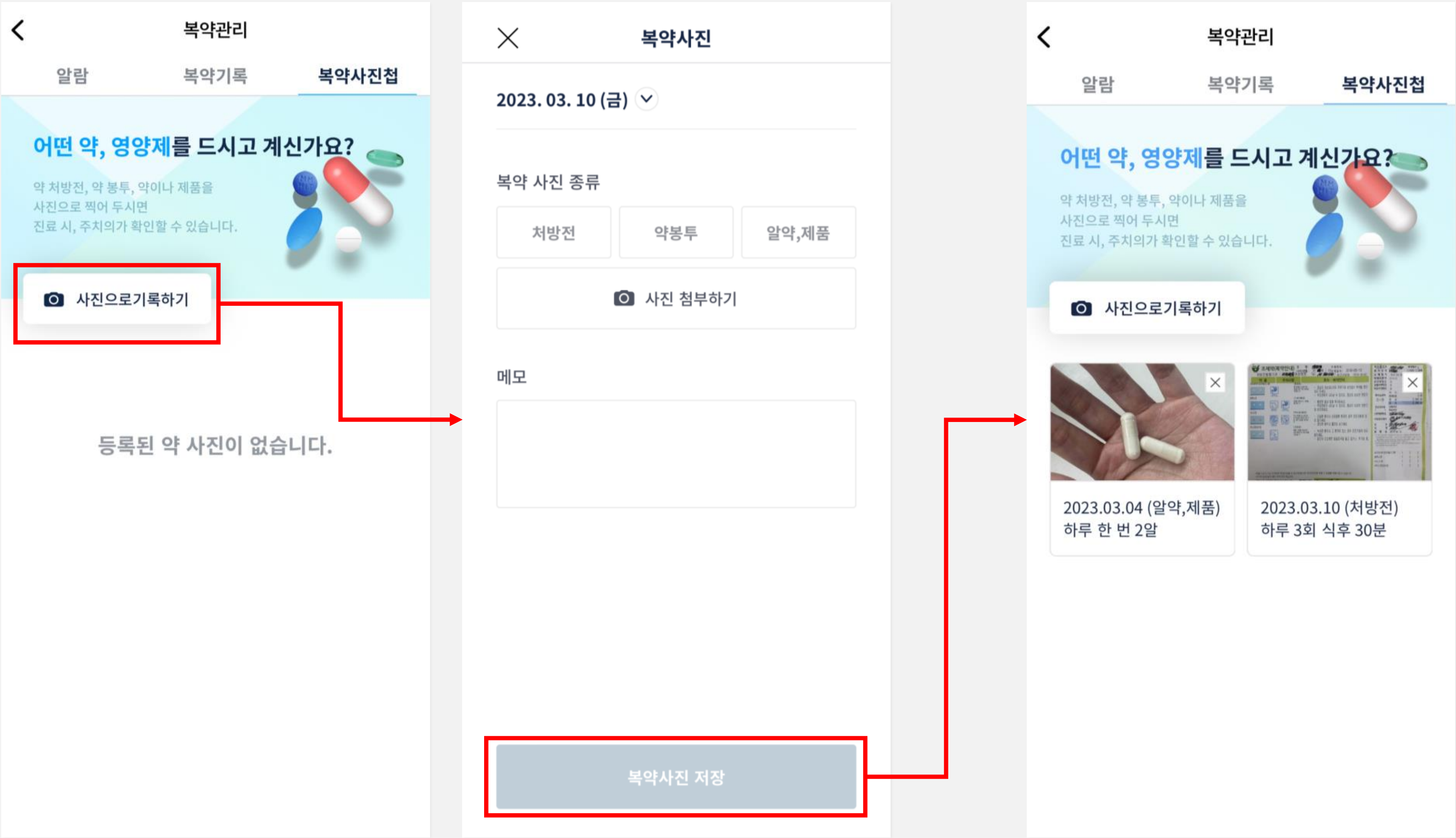

In the medication photo gallery tab, patients can also record medication using photos.

Patients can select the type of medication photo and easily enter a memo, and doctors can monitor the patient's medication history in detail.

[The process of using Well-check] Medication_Web

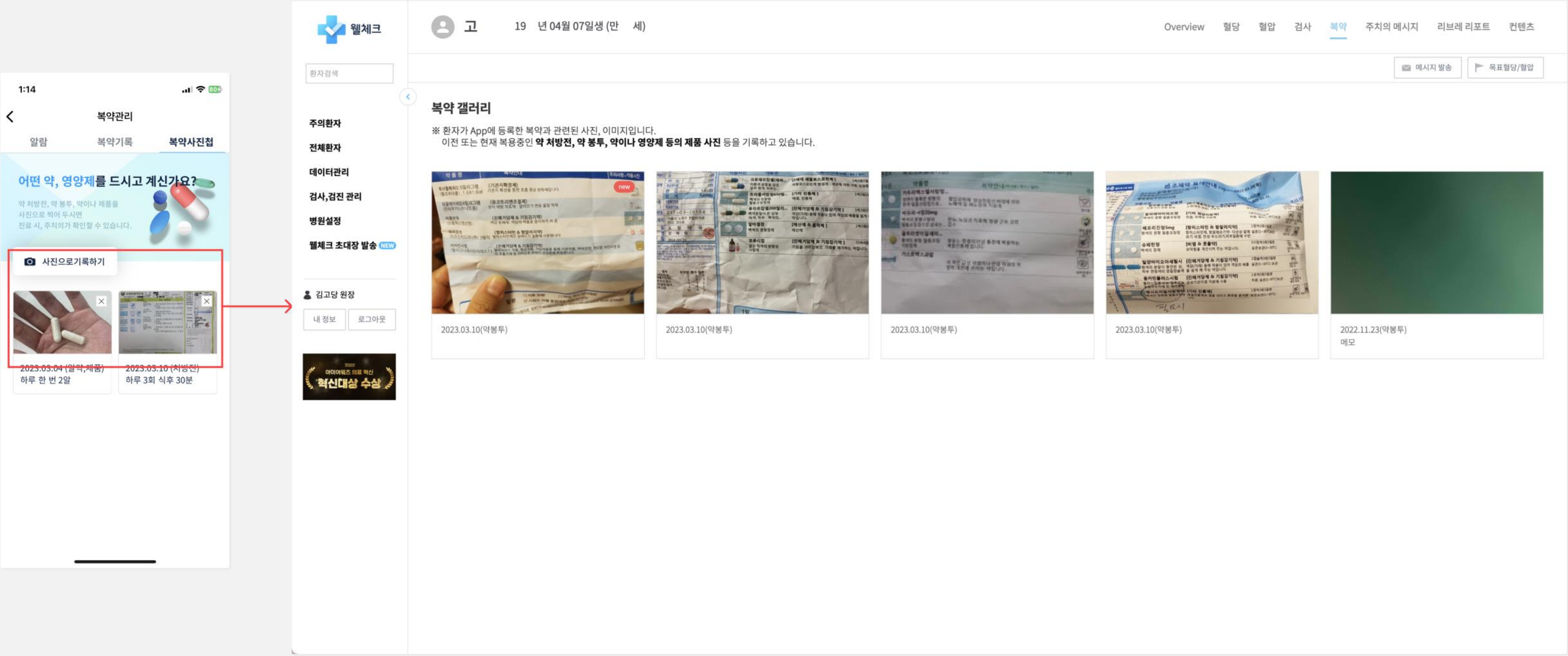

The doctor can assess whether the patient is taking their medication properly and, if not, what can be done to address it, based on the medication gallery, and can incorporate this into the treatment.

04 (App)

Etc.

The process of using Well check_App

- 1. Message from the doctor
- 2. Health information
- 3. Health screening test instructions

[The process of using Well-check] Message from the doctor_App

1

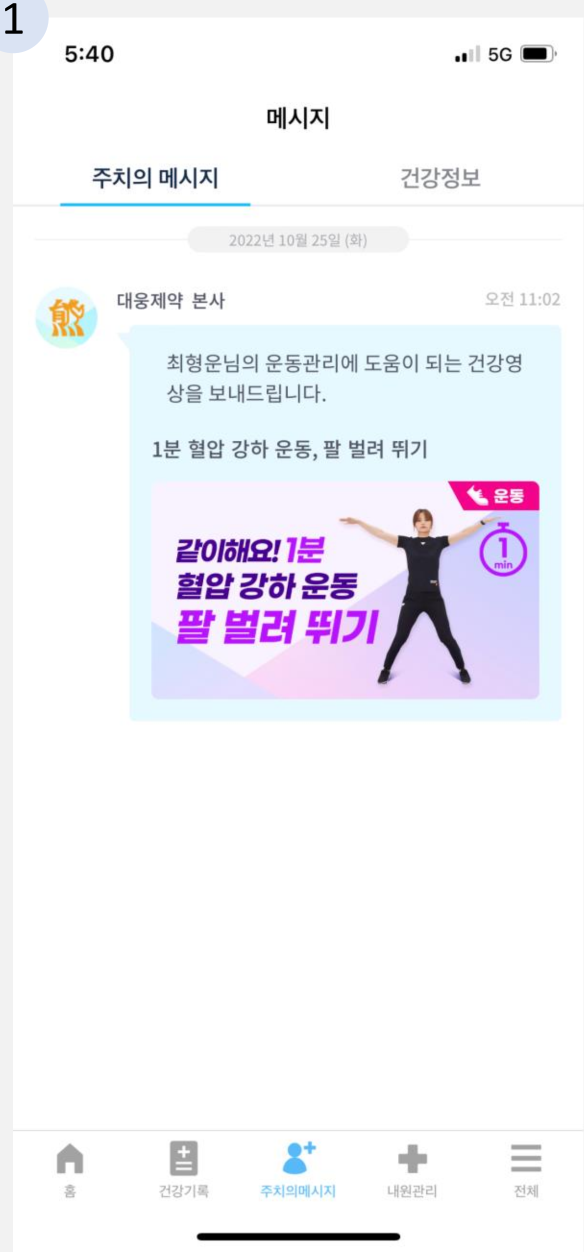

2

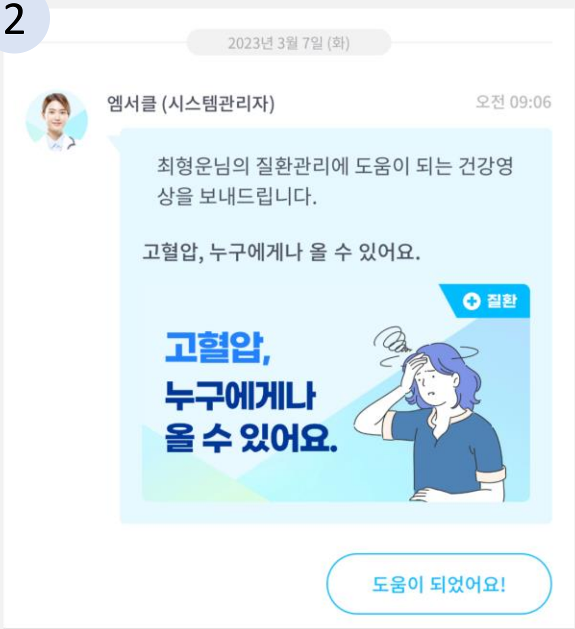

1

Patients can provide feedback on whether the doctor's messages were helpful through the button. Doctors can review this feedback and improve their messages accordingly.

2

The messages sent to the patient by the doctor are displayed in the app as a chat UI.

[The process of using Well-check] Health information_App

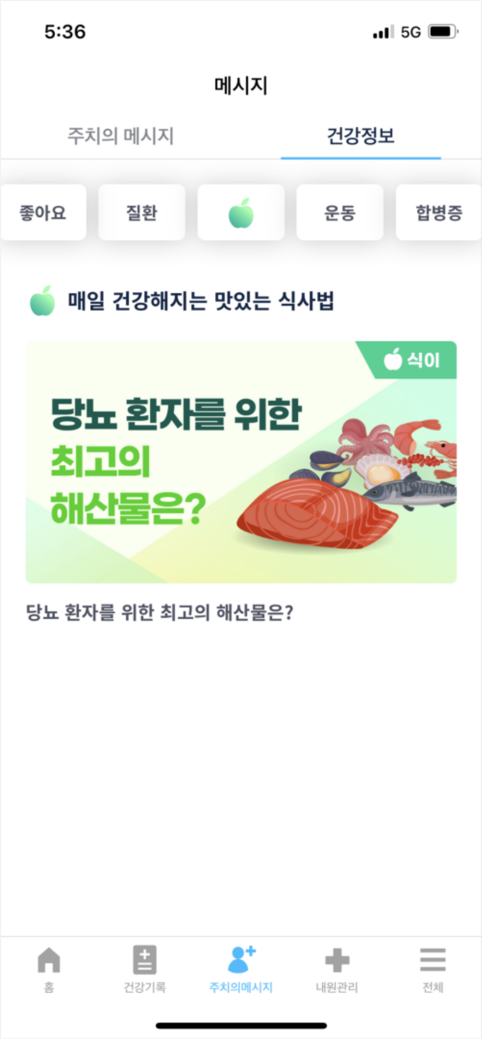

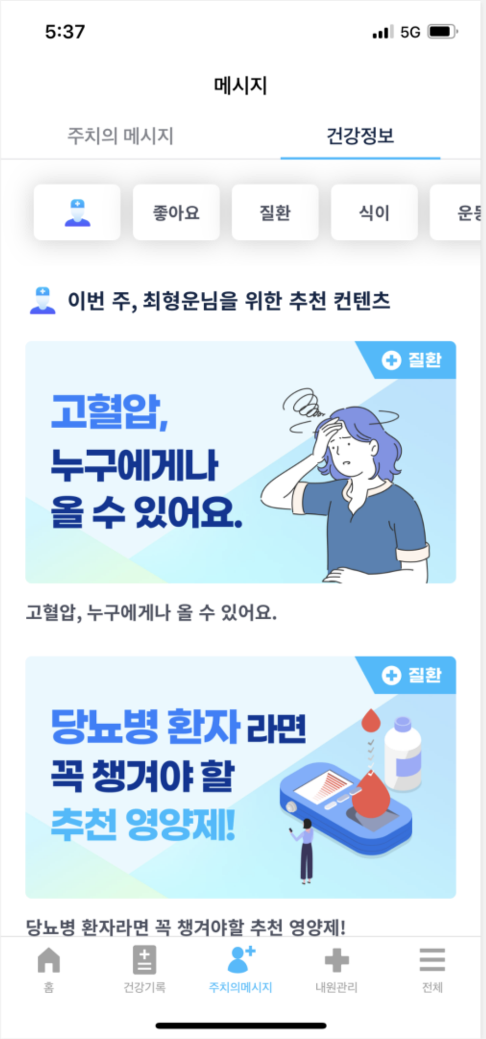

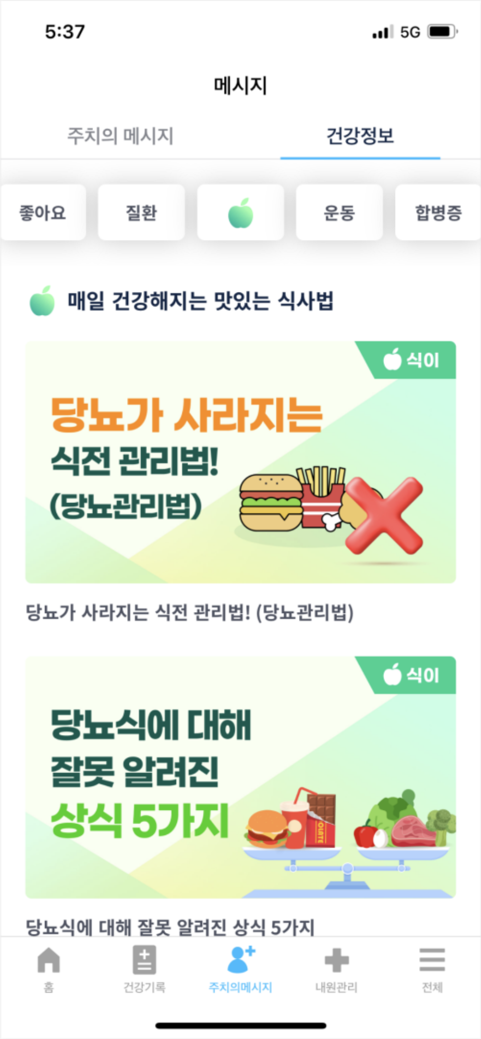

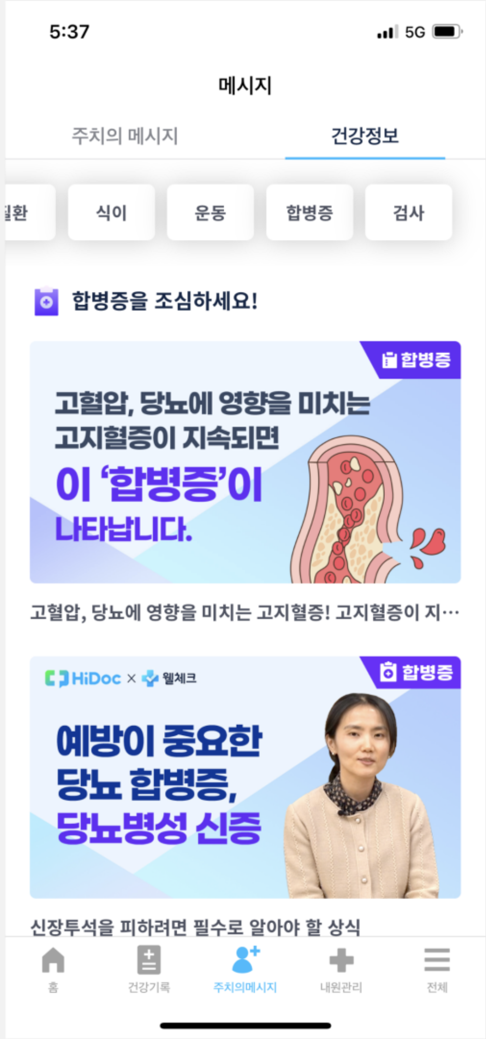

Well-check provides information helpful for managing chronic diseases, categorized and presented in a way that helps users easily learn the information they need.

[The process of using Well-check] Health screening instructions_App

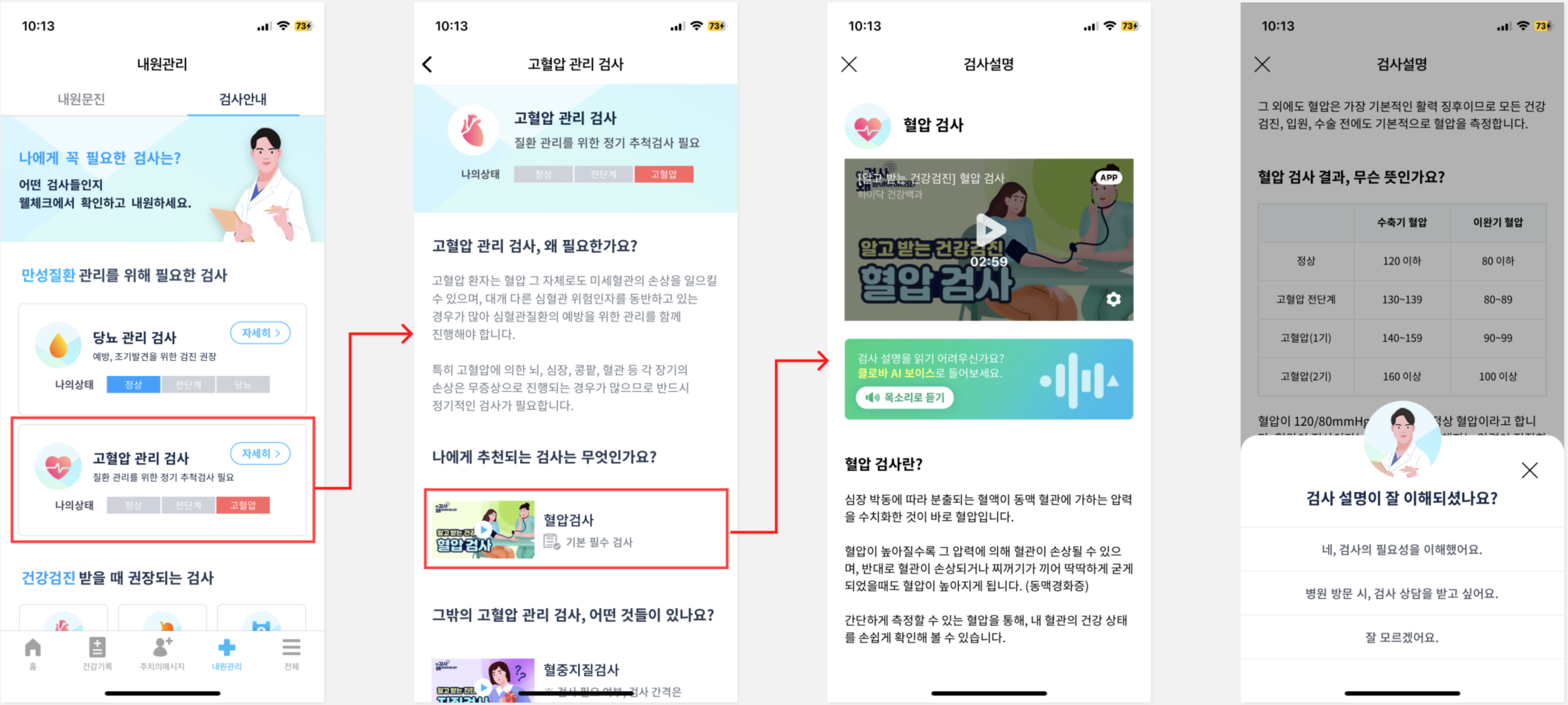

Patients are recommended personalized Health screening test tailored to their condition, and these tests are categorized by the patient's needs and provided to help them easily learn the information they want. The recommended Health screening test are also provided, along with reasons for the recommendations and precautions. After the education screen for the test is completed, patients can also check their understanding of the information and request consultation regarding the test.

01 (Web)

Blood pressure

The process of using Well-check_Web

[The process of using Well-check] Blood pressure_Web

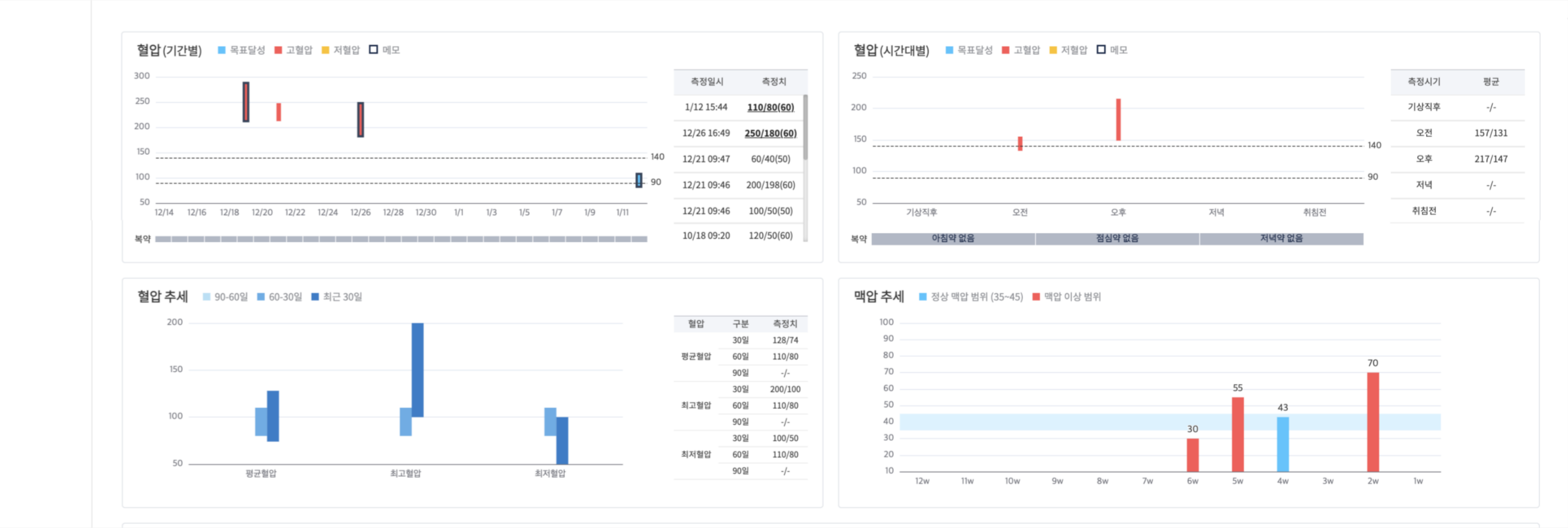

Doctors can receive analysis results on an individual patient's blood pressure trend, average, and degree of deviation from the target over the past 1-3 months, allowing them to understand the patient's condition and use it in treatment. They can also check the trend of whether the blood pressure is being well managed through a graph.

[The process of using Well-check] Blood pressure_Web

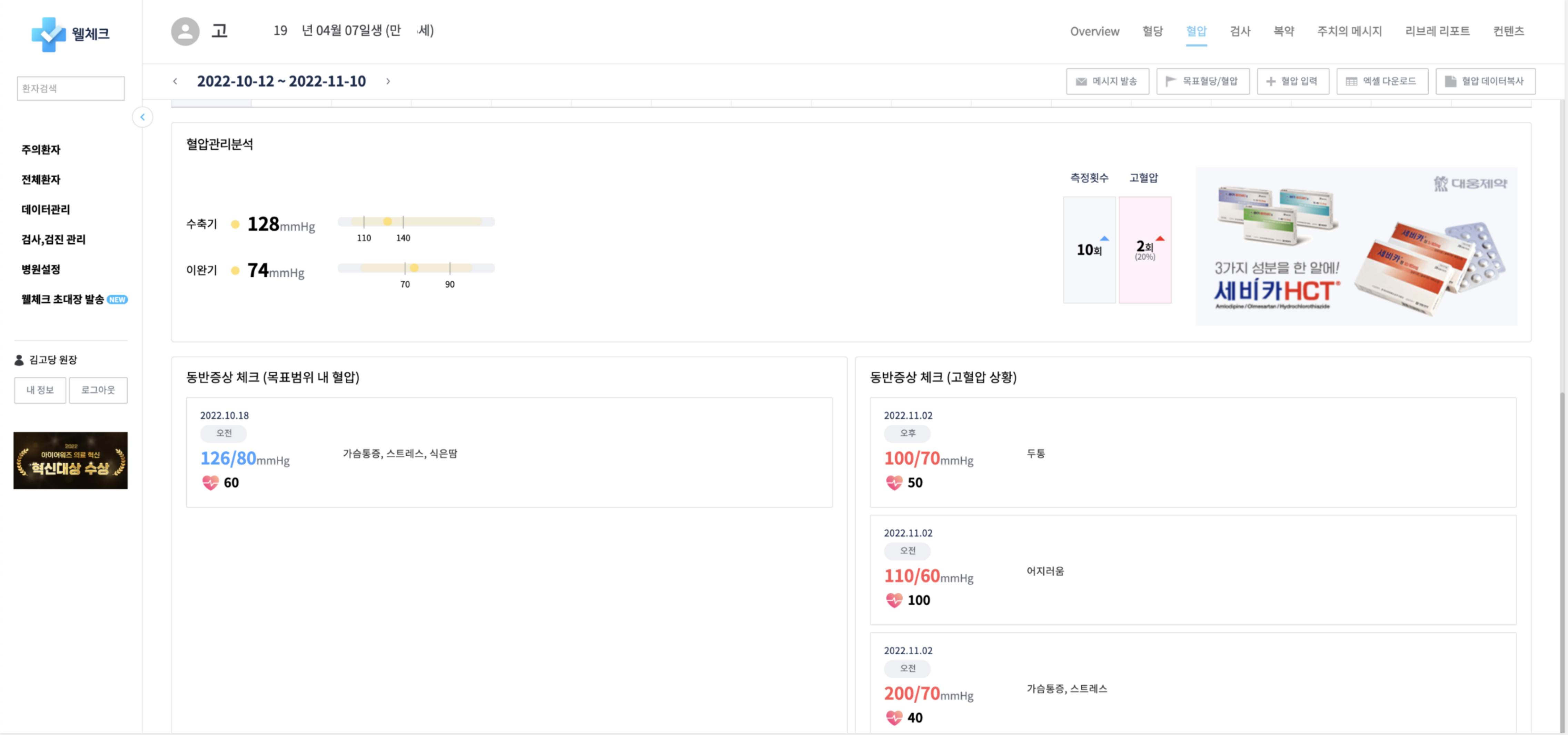

Doctors can check the details of accompanying symptoms when the patient recorded their blood pressure and left a memo.

02 (Web)

Blood sugar

The process of using Well-check_Web

[The process of using Well-check] Blood sugar_Web

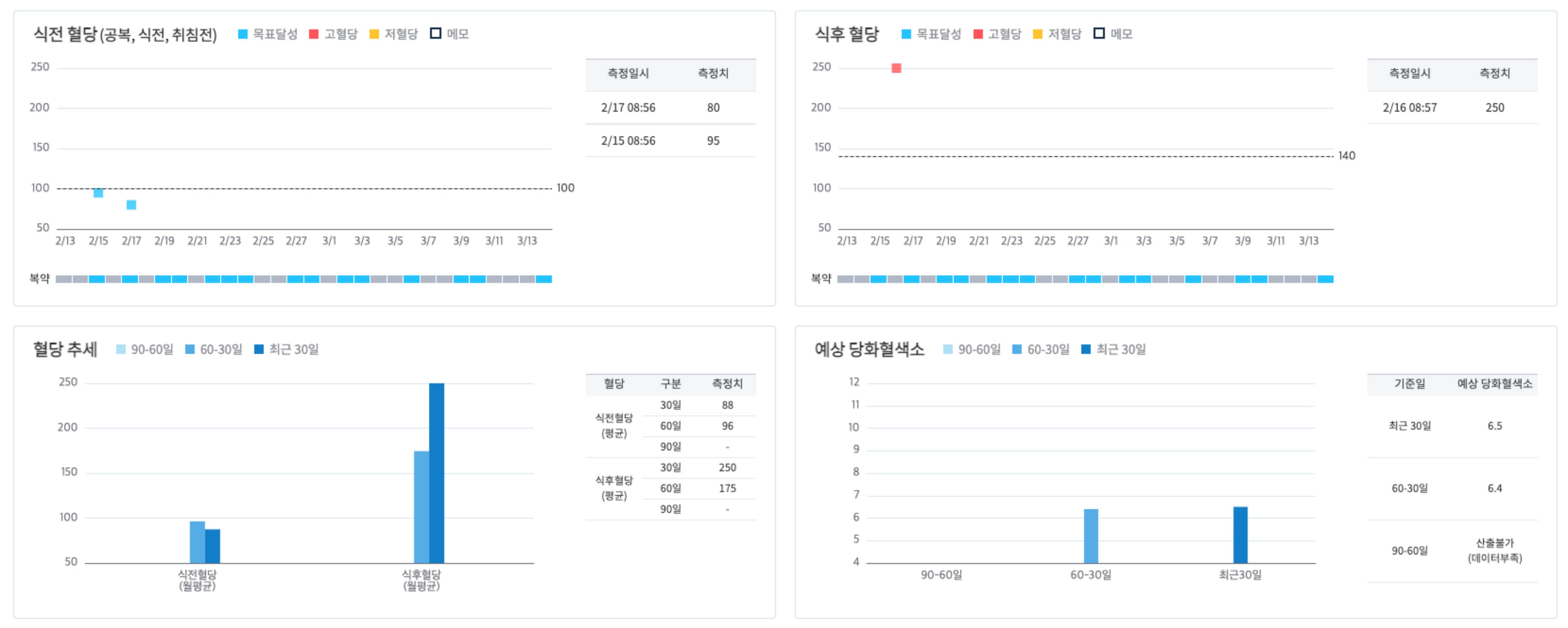

Along with medication status, pre/postprandial blood glucose levels are displayed, so doctors can also check the extent of blood glucose levels according to medication. In addition, they can check the recent 1-3 month trend of blood glucose level and expected HbA1c level.

[The process of using Well-check] Blood sugar_Web

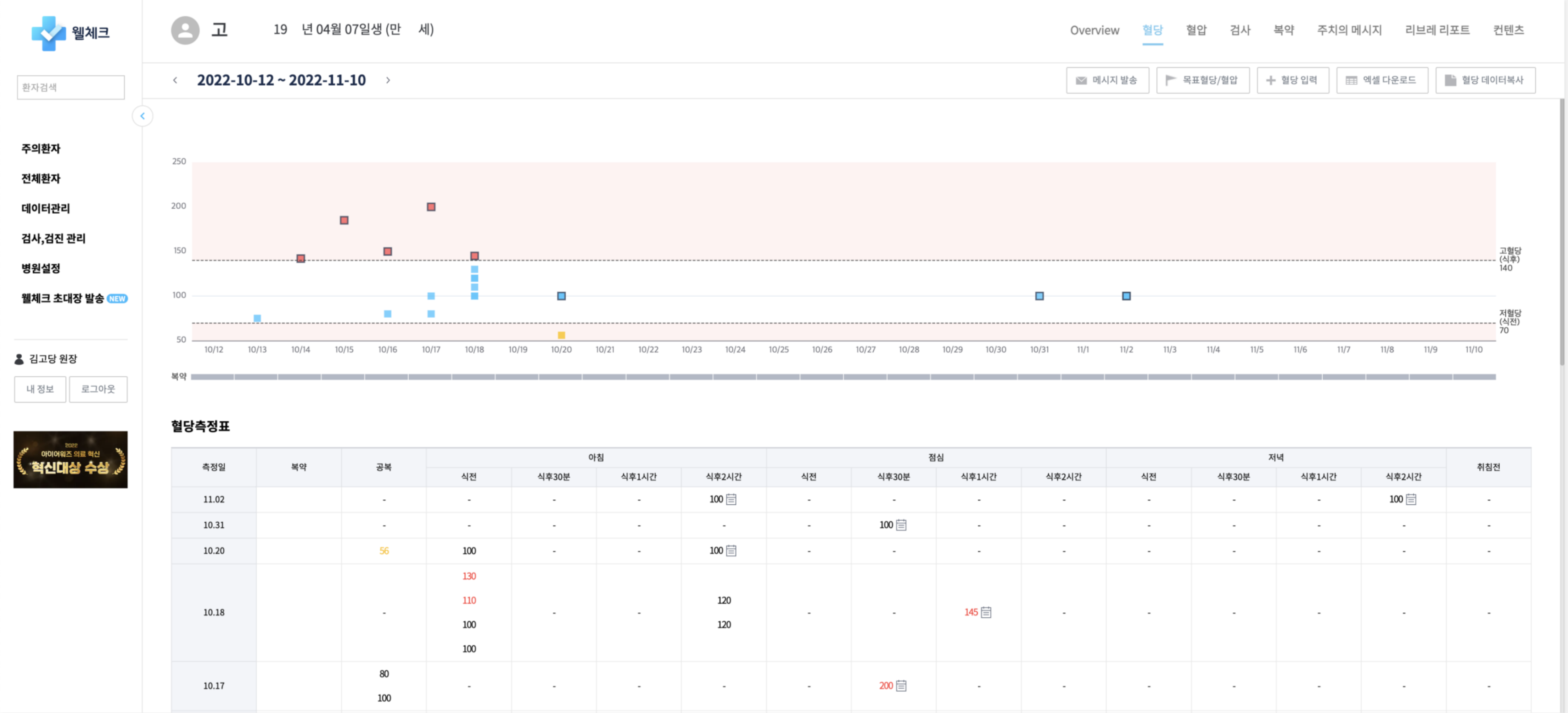

The doctor can receive an analysis of an individual patient's blood sugar trends, average, and how much they deviate from the target range over the past 1-3 months, and can use this information to assess the patient's condition and utilize it in treatment. They can also visually confirm the trend of blood sugar management through graphs.

[The process of using Well-check] Blood sugar_Web

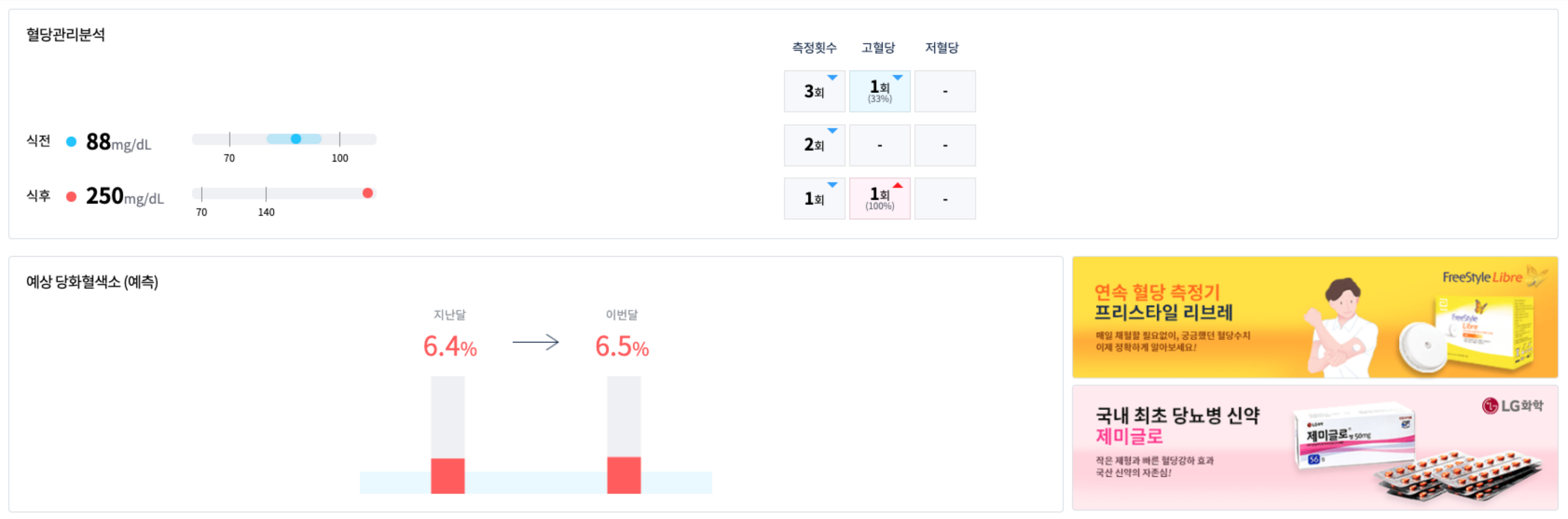

The doctor can check the frequency of hyperglycemia and hypoglycemia according to the number of measurements, and can also grasp the approximate range of pre/postprandial blood glucose levels. In addition, they can refer to the previous month's HbA1c level and check the predicted HbA1c level for this month.

[The process of using Well-check] Blood sugar_Web

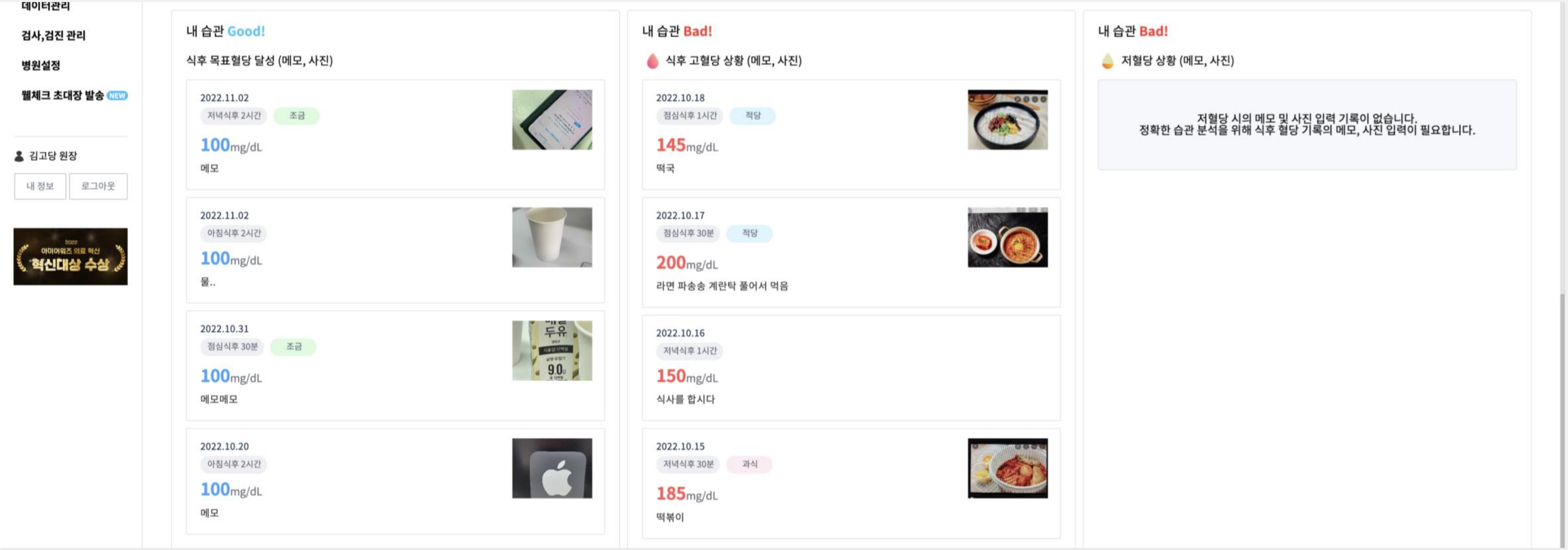

The doctor can check what food the patient consumed when their blood glucose was in the normal range, and what food they consumed and any notes they left when their blood glucose was high or low.

03 (Web)

Medication

The process of using Well-check_Web

[The process of using Well-check] Medication_Web

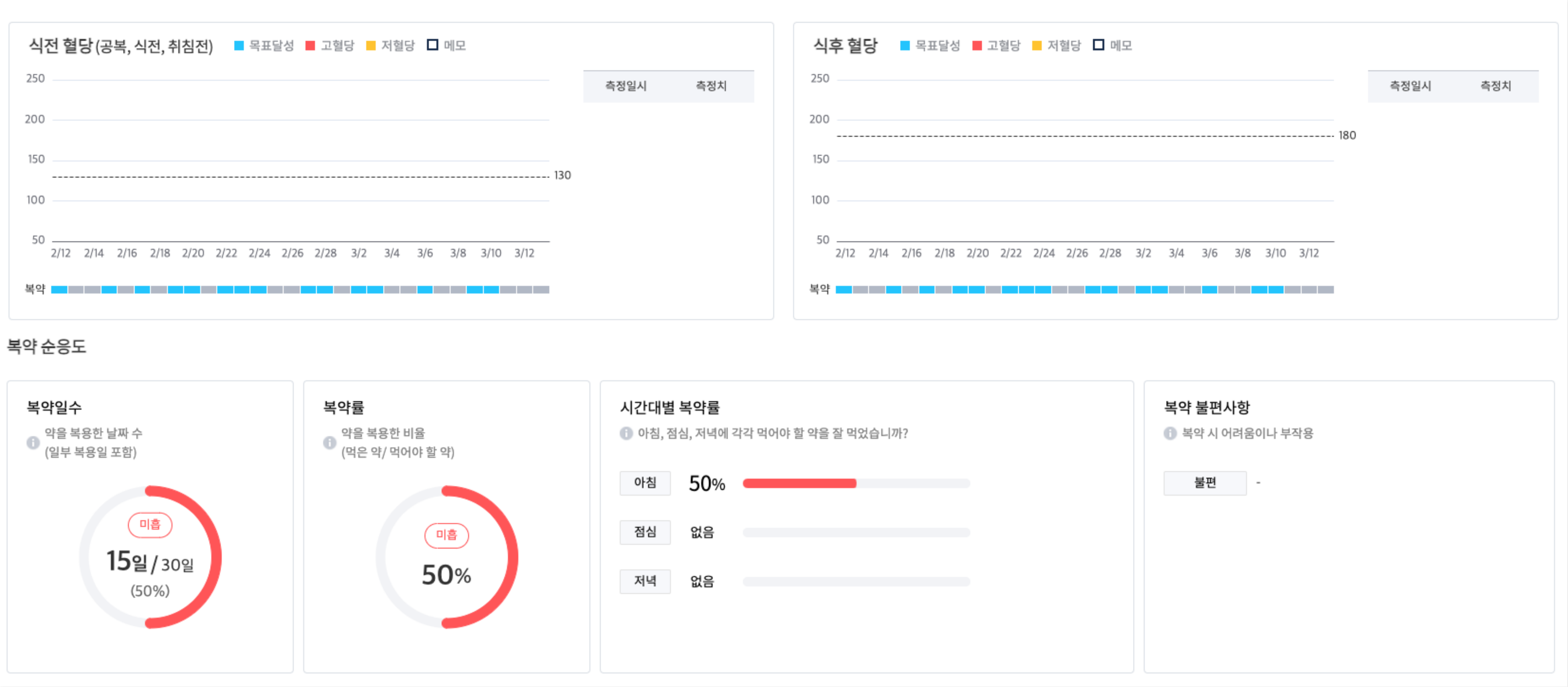

Medication adherence is analyzed in detail by the number and frequency of medication days, as well as the timing of medication (morning/noon/evening), and any discomfort related to medication is also displayed.

04 (Web)

Etc.

The process of using Well check_Web

- 1. Message from the doctor
- 2. Health screening test instructions
- 3. High-risk patient monitoring

[The process of using Well-check] Message from the doctor_Web_Check sent messages

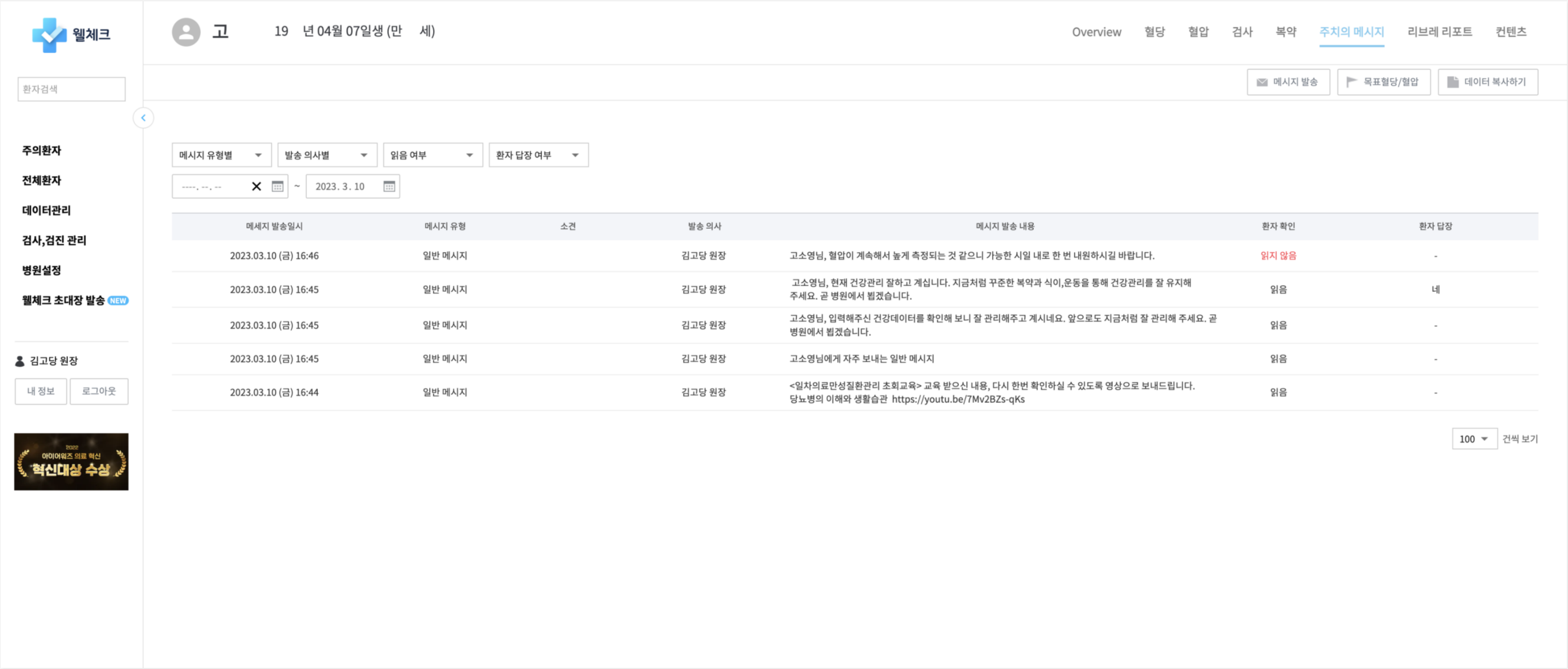

Doctors can also check whether patients have viewed and responded to messages in the message history.

[The process of using Well-check] Message from the doctor_Web_Message settings

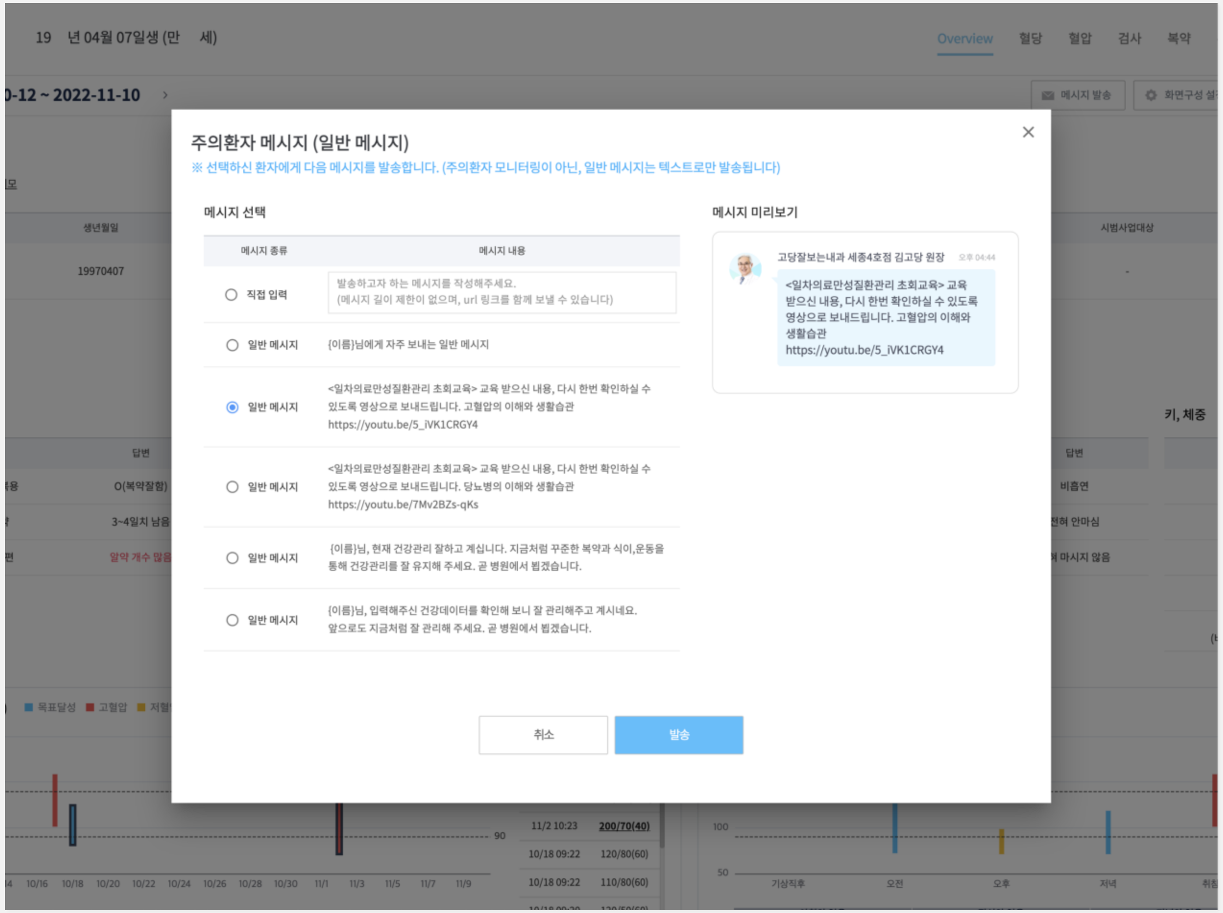

Doctors can send educational content to patients through "message sending" for patients who are poorly managed or need special attention, and guide them to visit the hospital for treatment.

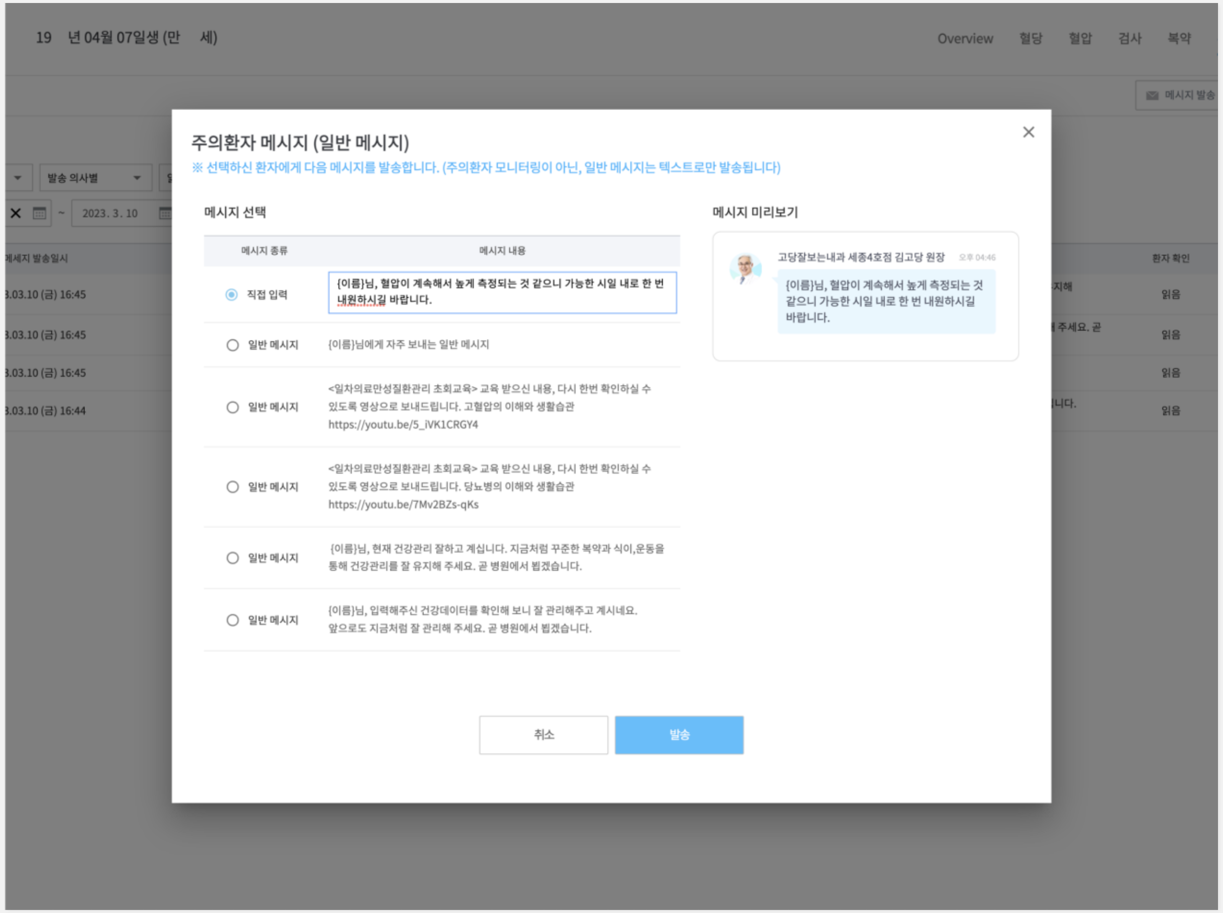

When doctors write {name} in the message content, it is automatically applied with the patient's name when sending the message, so they can send a more friendly message.

[The process of using Well-check] Health screening test instructions_Settings _Web

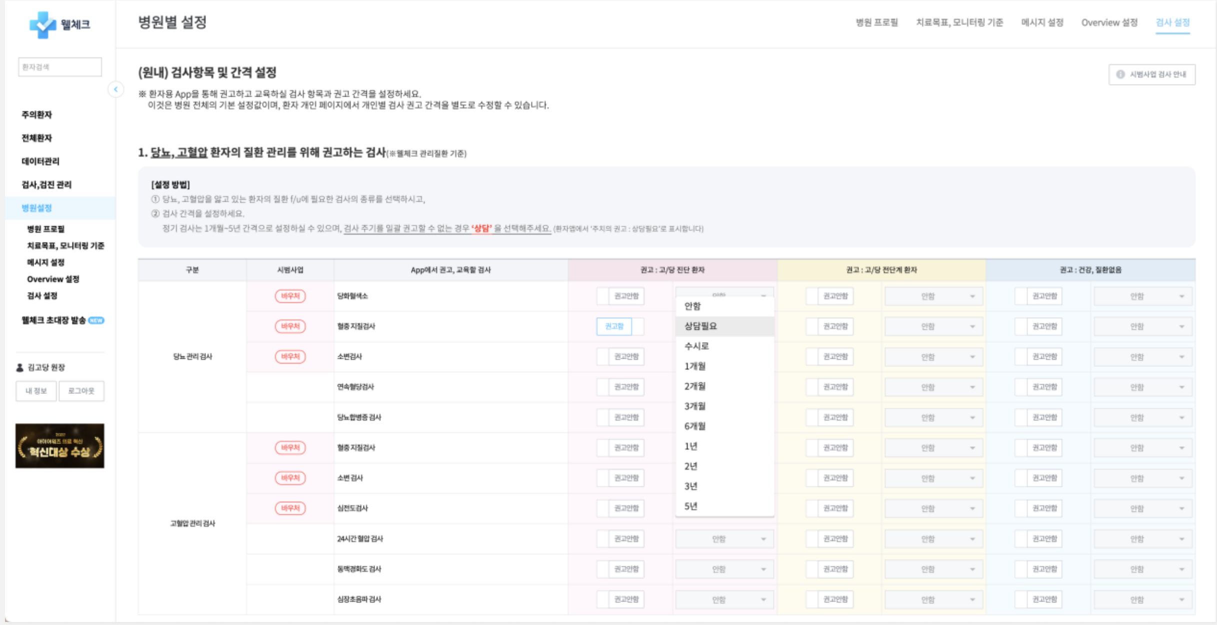

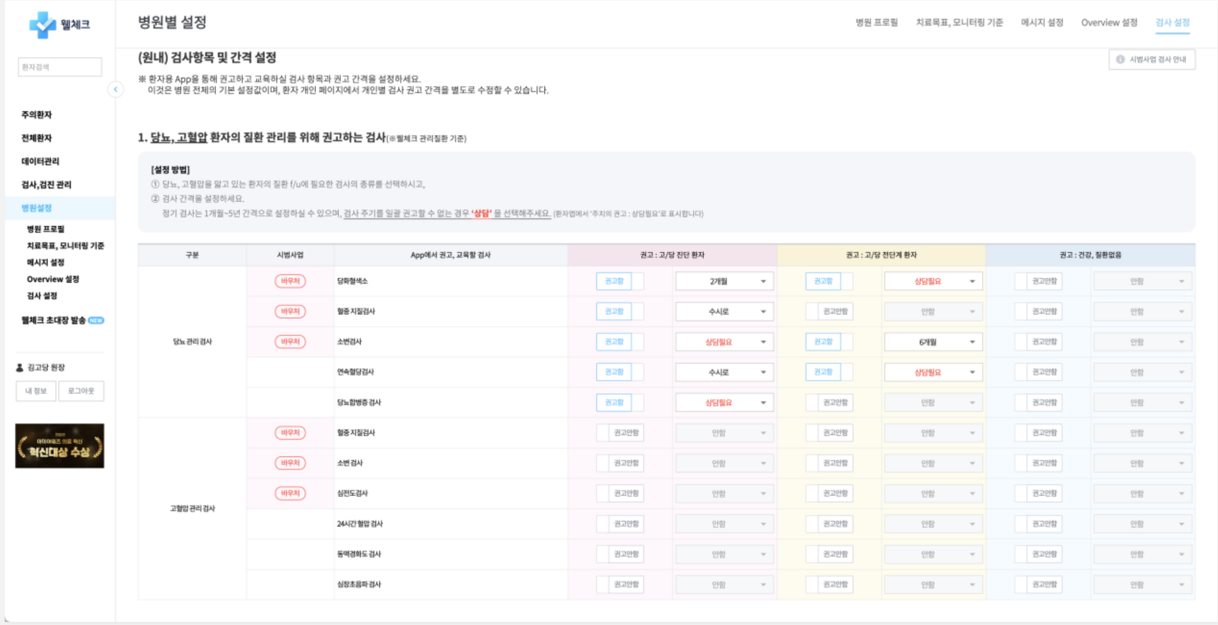

Doctors can recommend tests based on the patient's current medical condition and automatically recommend the test frequency to the patient according to the settings on the doctor's web page.
(Recommended tests include those needed for managing diabetes and hypertension as well as cardiovascular, digestive, and other health screenings.)

[The process of using Well-check] Health screening test instructions_Checking _Web

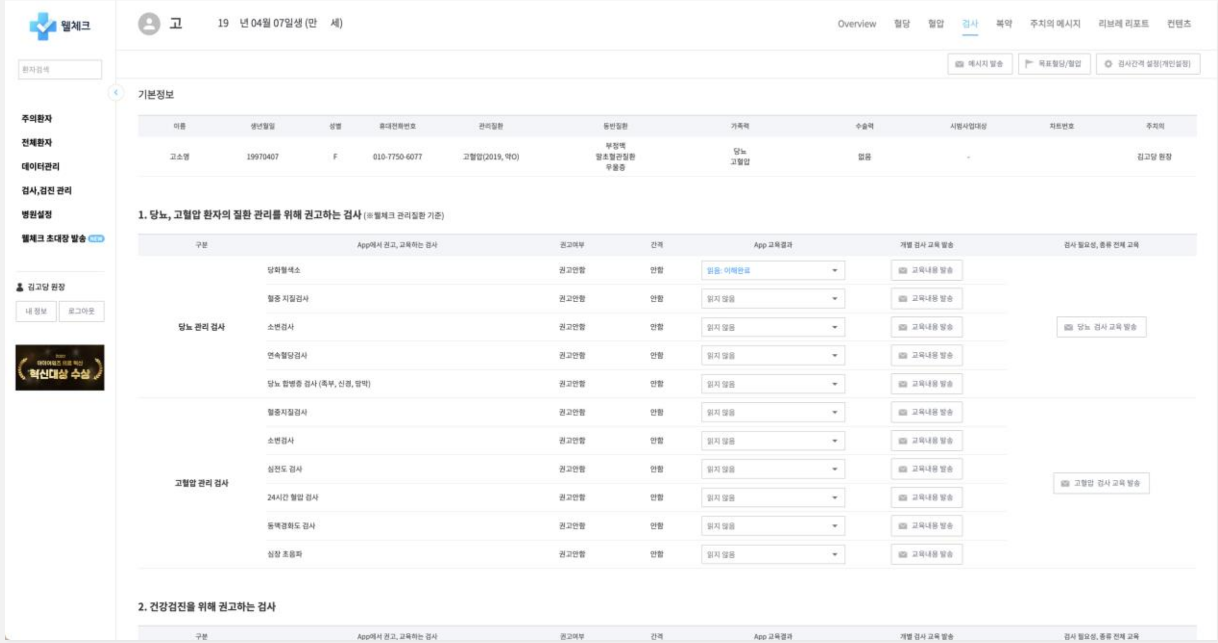

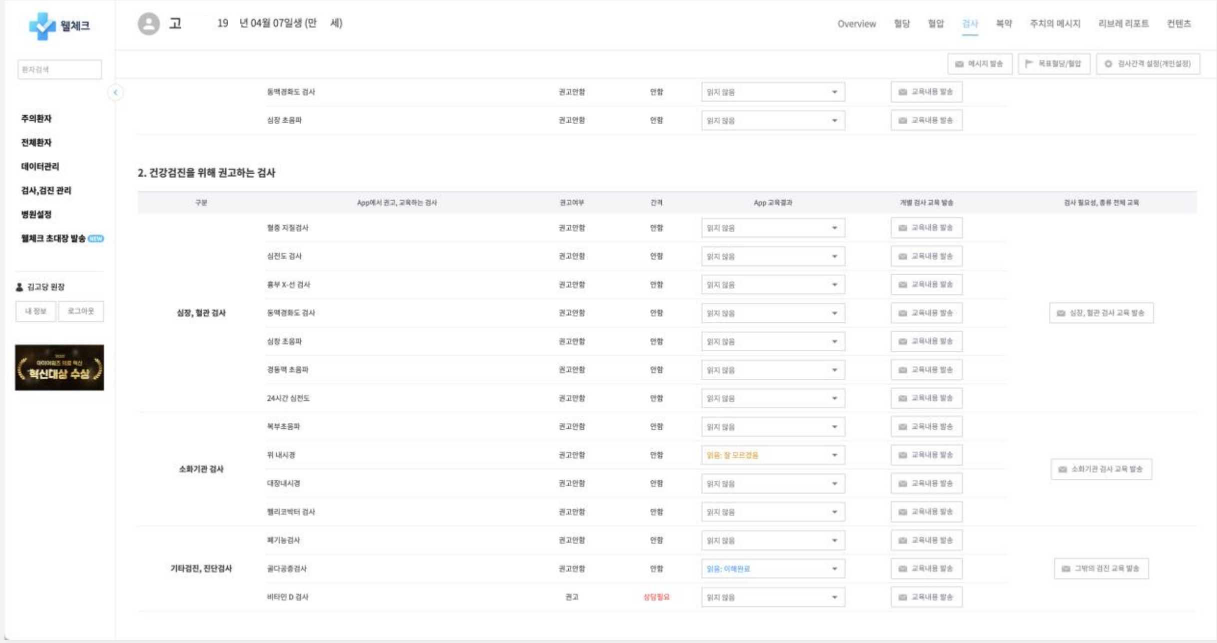

Doctors can identify the type of education the patient has received, whether the patient has read the education materials, and whether the patient wants counseling, not just the degree of understanding. If the patient does not understand the information well enough, doctors can easily resend the explanation of the test to the patient with just one click of a message.

[The process of using Well-check] Health screening test instructions _View patient test/examination results _Web

웰체크

환자검색

주의환자
전체환자
데이터관리
검사,검진 관리
병원설정
웰체크 초대장 발송 NEW

김고당 원장

내 정보 | 로그아웃

2022 아이어워즈 의료 혁신 혁신대상 수상

검사,검진 관리

다운로드

검진 내역 다운로드

연도

2021

포함 내역

☑ 생년월일 ☑ 휴대전화 ☑ 주치의

제공 검진 내역: 공복혈당
수축기혈압, 이완기혈압
총콜레스테롤, HDL콜레스테롤, 중성지방, LDL콜레스테롤
신장, 체중, 허리둘레, 체질량지수
혈청크레아티닌, 신사구체여과율
요단백
AST(SGOT), ALT(SGPT), 감마지피티(y-GTP)
혈색소
시력(좌/우), 청력(좌/우)
폐결핵 흉부질환
골다공증

이름	생년월일	휴대전화번호	주치의	공복혈당 (mg/dL)	수축기혈압 (mmHg)	이완기혈압 (mmHg)	총콜레스테롤 (mg/dL)	HDL콜레스테롤 (mg/dL)	중성지방 (mg/dL)	LDL콜레스테롤 (mg/dL)	신장 (cm)	체중 (kg)	허리둘레 (cm)	체질량지수 (kg/m2)	혈청크레아티닌 (mg/dL)	신사구체여과율 (mL/min)	요단백	AST(SGOT) (U/L)	ALT(SGPT) (U/L)	감마지티피(y-GTP) (U/L)	혈색소 (g/dL)	시력 (좌)	시력 (우)	청력 (좌)	청력 (우)	폐결핵 흉부질환	골다공증 (T-score)
김 롱	19 0425	8210 8589	김고당 원장	97	112	71	-	-	-	-	161.9	59.6	75.0	22.7	0.5	146	음성	13	9	11	12.5	1.0	1.0	정상	정상	정상	-
유 훈	19 0807	8210 1277	김고당 원장	90	134	88	-	-	-	-	171.2	79.3	93.0	27.1	0.8	96	음성	38	64	31	15.0	0.8	0.7	정상	정상	정상	-

100 건씩 보기

Doctors can check and download their patients' health checkup records that they manage.

[The process of using Well-check] High-risk patient monitoring_Web

1. High-risk patient monitoring _ Setting blood sugar/blood pressure target and caution criteria.

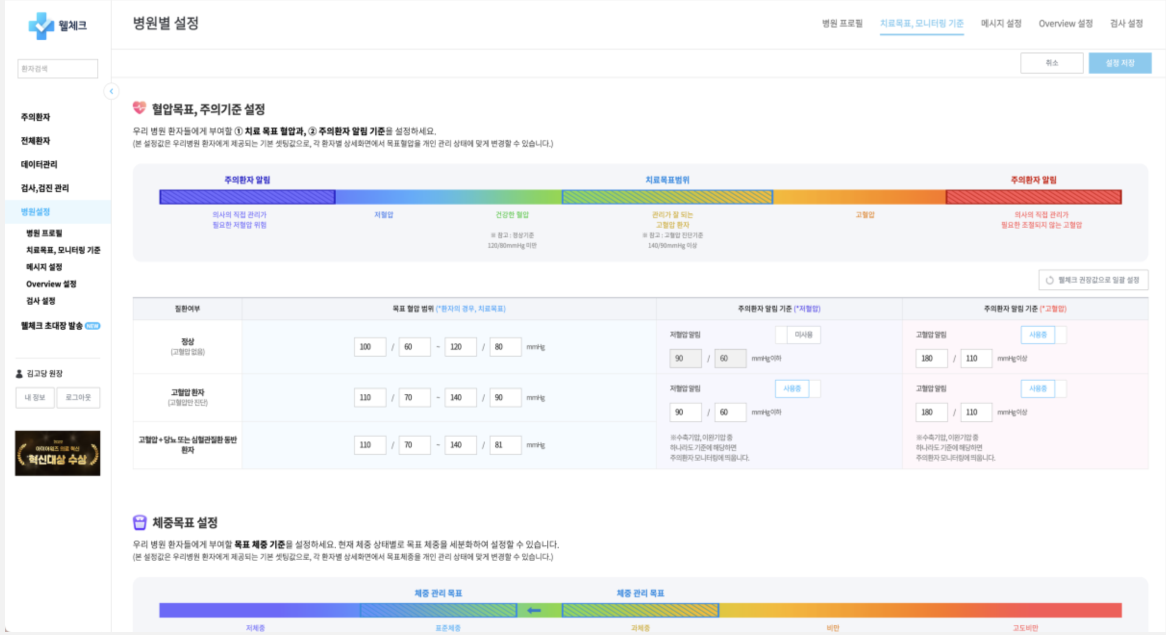

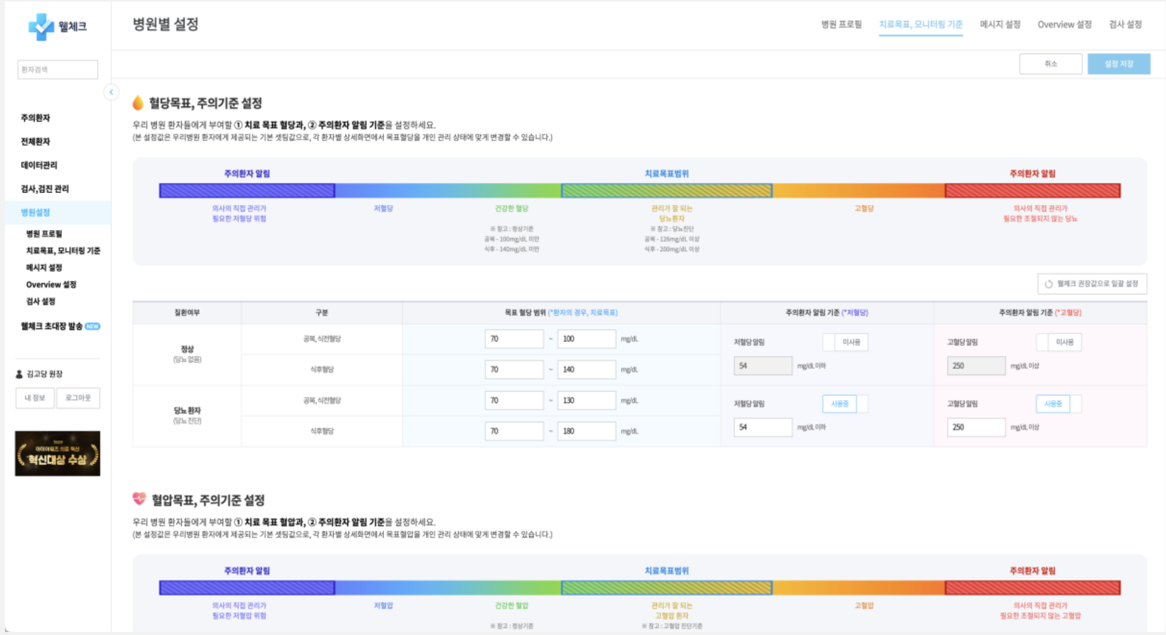

Doctors can set target ranges for blood sugar and blood pressure, as well as input warning thresholds for blood sugar and blood pressure for high-risk patients.

[The process of using Well-check] High-risk patient monitoring_Web

1. High-risk patient monitoring _ Identification and message guidance for high-risk patients.

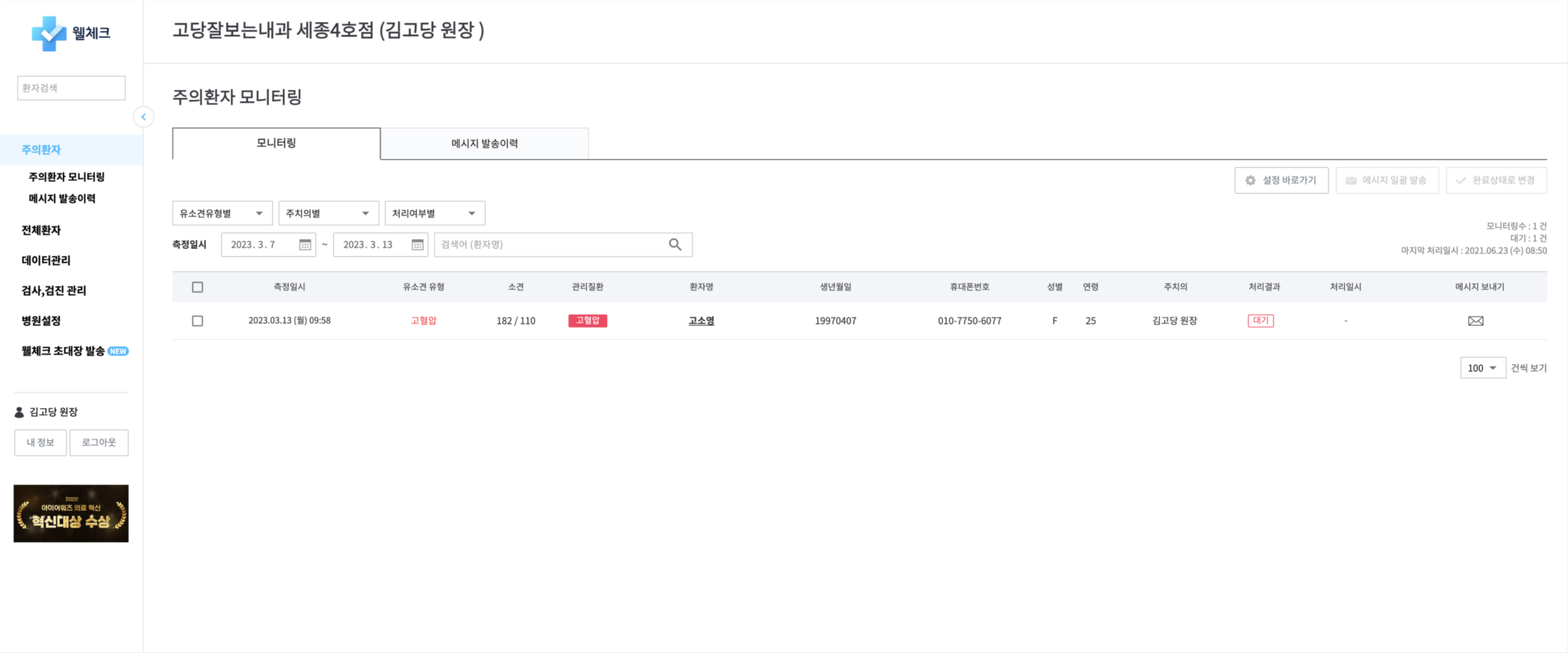

If there is an abnormal blood pressure or blood sugar level that exceeds the alert criteria for at-risk patients, a notification will be generated in the at-risk patient monitoring list. Through this, doctors can monitor at-risk patients separately and view message sending history together.
