# Supplementary figures and images for: Real-World Evidence of a Hospital-Linked Digital Health App for the Control of Hypertension and Diabetes Mellitus in South Korea: Nationwide Multicenter Study
Source: JMIR Form Res. 2023 Aug 21;7:e48332. doi: 10.2196/48332 (PMC10477930; doi:10.2196/48332)

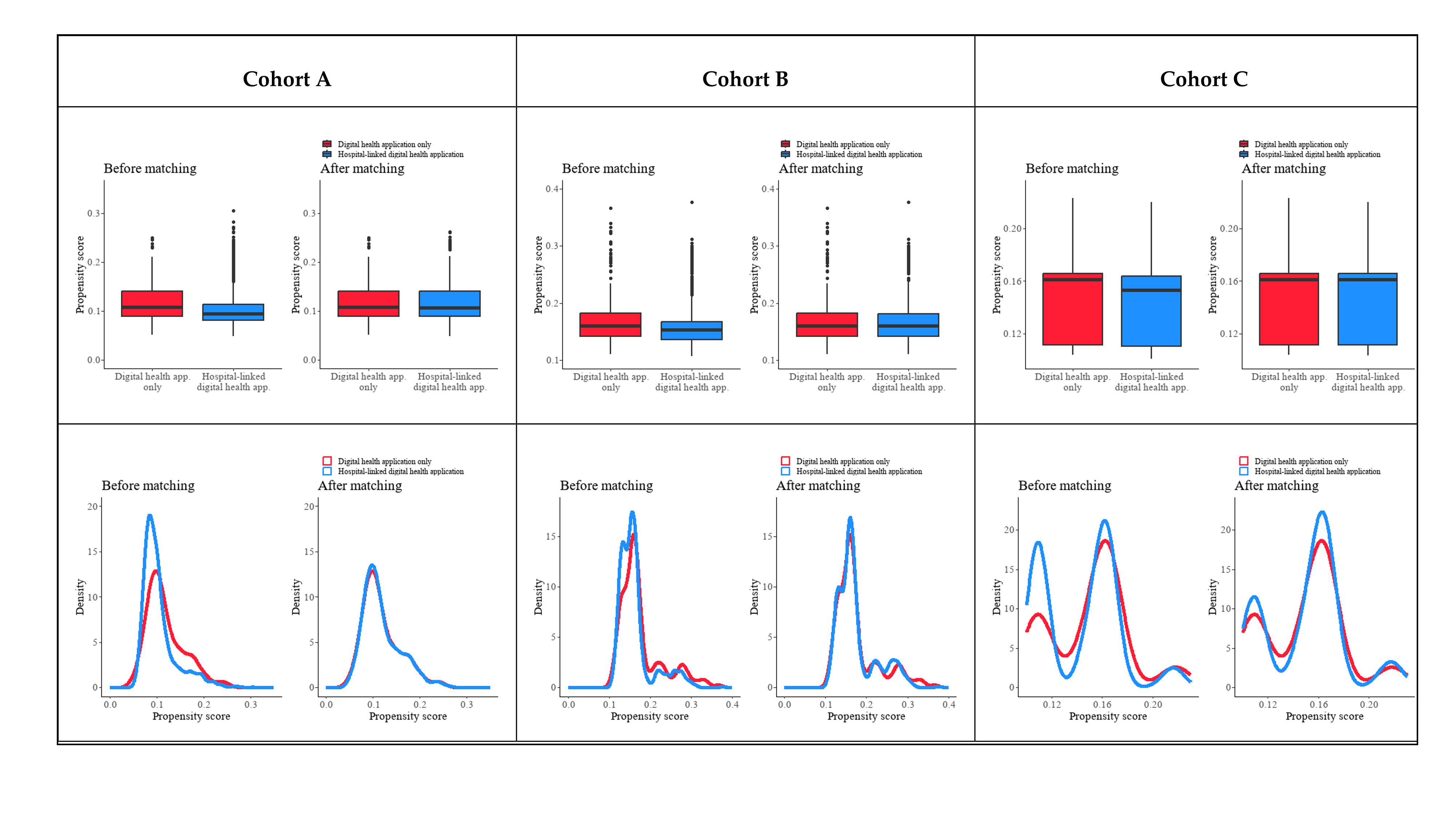

Supplement: Multimedia Appendix 2 [file formative_v7i1e48332_app2.zip › F2_2.png]
